# Molecular and functional signatures in a novel Alzheimer’s disease mouse model assessed by quantitative proteomics

**DOI:** 10.1186/s13024-017-0234-4

**Published:** 2018-01-16

**Authors:** Dong Kyu Kim, Joonho Park, Dohyun Han, Jinhee Yang, Ahbin Kim, Jongmin Woo, Youngsoo Kim, Inhee Mook-Jung

**Affiliations:** 1 Department of Biomedical Sciences, Seoul National University, College of Medicine, 103 Daehak-ro, Seoul, 110-799 South Korea; 2 Interdisciplinary Program in Bioengineering, College of Engineering, Seoul National University, 1 Gwanak-ro, Seoul, 151-742 South Korea; 3 Biomedical Research Institute, Seoul National University Hospital, 101 Daehak-ro, Seoul, 110-744 South Korea; 4 Neuroscience Research Institute, Seoul National University, College of Medicine, Seoul, 110-799 South Korea

**Keywords:** Alzheimer’s disease, Animal disease model, Aβ, Tau, Quantitative proteomics, 10-plex tandem mass tag

## Abstract

**Background:**

Alzheimer’s disease (AD), the most common neurodegenerative disorder, is characterized by the deposition of extracellular amyloid plaques and intracellular neurofibrillary tangles. To understand the pathological mechanisms underlying AD, developing animal models that completely encompass the main features of AD pathologies is indispensable. Although mouse models that display pathological hallmarks of AD (amyloid plaques, neurofibrillary tangles, or both) have been developed and investigated, a systematic approach for understanding the molecular characteristics of AD mouse models is lacking.

**Methods:**

To elucidate the mechanisms underlying the contribution of amyloid beta (Aβ) and tau in AD pathogenesis, we herein generated a novel animal model of AD, namely the AD-like pathology with amyloid and neurofibrillary tangles (ADLP^APT^) mice. The ADLP^APT^ mice carry three human transgenes, including amyloid precursor protein, presenilin-1, and tau, with six mutations. To characterize the molecular and functional signatures of AD in ADLP^APT^ mice, we analyzed the hippocampal proteome and performed comparisons with individual-pathology transgenic mice (i.e., amyloid or neurofibrillary tangles) and wild-type mice using quantitative proteomics with 10-plex tandem mass tag.

**Results:**

The ADLP^APT^ mice exhibited accelerated neurofibrillary tangle formation in addition to amyloid plaques, neuronal loss in the CA1 area, and memory deficit at an early age. In addition, our proteomic analysis identified nearly 10,000 protein groups, which enabled the identification of hundreds of differentially expressed proteins (DEPs) in ADLP^APT^ mice. Bioinformatics analysis of DEPs revealed that ADLP^APT^ mice experienced age-dependent active immune responses and synaptic dysfunctions.

**Conclusions:**

Our study is the first to compare and describe the proteomic characteristics in amyloid and neurofibrillary tangle pathologies using isobaric label-based quantitative proteomics. Furthermore, we analyzed the hippocampal proteome of the newly developed ADLP^APT^ model mice to investigate how both Aβ and tau pathologies regulate the hippocampal proteome. Because the ADLP^APT^ mouse model recapitulates the main features of AD pathogenesis, the proteomic data derived from its hippocampus has significant utility as a novel resource for the research on the Aβ-tau axis and pathophysiological changes in vivo.

**Electronic supplementary material:**

The online version of this article (10.1186/s13024-017-0234-4) contains supplementary material, which is available to authorized users.

## Background

Alzheimer’s disease (AD) is the most common neurodegenerative disorder and is characterized by extracellular amyloid plaque deposition, intracellular neurofibrillary tangle (NFT) development, and memory impairments [1, 2]. Although the disease has been extensively studied over the past several decades, the exact mechanisms and pathological causes of AD remain unclear. Genetically modified mouse models that recapitulate the major features of AD pathologies are invaluable in determining the underlying disease mechanisms and evaluating new therapeutic approaches. Few transgenic mouse models, such as the triple transgenic mice, 3xTg-AD model, have been introduced to develop the concomitant manifestation of both amyloid plaques and NFT formation [3]. Because this model showed no neuronal cell death in the hippocampus, the development of a new mouse model that fully mimics AD pathologies is still needed.

One of the most widely used models in AD research is the 5xFAD (Tg6799) model, which contains five familial AD mutations within the *APP* and *PSEN1* genes. The 5xFAD model is considered to be an effective AD model because of the rapid progression of the amyloid pathology [4]. The JNPL3 mouse model, which expresses P301L-mutant human tau, has been widely used to examine intraneuronal NFTs [5]. To recapitulate the main features of AD pathogenesis, we herein developed a new mouse model that carries six mutations within transgenes encoding human amyloid precursor protein (APP), presenilin-1 (PSEN1), and tau. The resulting transgenic mouse model, Alzheimer’s disease-like pathology with *APP*, *PSEN1,* and *MAPT* transgenes (ADLP^APT^), exhibited Aβ accumulation, NFTs, early neuronal loss in the brain, and subsequent memory impairments. The pathological phenotypes of ADLP^APT^ mice, which feature both Aβ deposition and NFTs, were compared with those of ADLP^APP/PS1^ (Aβ deposition, no NFTs) and ADLP^Tau^ (no Aβ deposition, NFTs) mice of the same genetic background. Understanding the interplay between Aβ accumulation and NFTs is imperative for elucidating the pathogenesis of AD. Thus, the ADLP^APT^ mouse model, which shows both robust amyloid and NFT pathologies, should be an excellent model for examining the Aβ-tau axis in vivo.

The hippocampus is known to play an important role in memory formation [6]. Thus, understanding the pathological status of the hippocampus under AD is crucial for studying the mechanisms of AD-related memory impairments. Although some mechanisms that contribute to AD pathogenesis have been uncovered by studies on individual genes or proteins, systematic analysis of the pathological changes of the hippocampus is lacking. Mass spectrometry (MS)-based proteomics is expected to be an appropriate tool for systematic analysis [7, 8]. Although MS-based proteomics have been limited because of the incomplete coverage of the proteome, recent technological advances allowed researchers to study comprehensively up to 10,000 proteins from a single cell line [9]. However, this level of coverage requires extensive pre-fractionation, large samples, and several months of instrument time [10]. In addition, the reliable MS-based quantitation under several perturbation states requires the use of biological and technical replicates, thus increasing the complexity of MS experiments. Importantly, these shortcomings can be overcome by an isobaric labeling strategy, such as the application of tandem mass tags (TMTs) [11]. The recent expansion of multiplexing capacity up to 10 samples per MS injection has markedly increased the scope of quantitative proteomics [12]. In TMT experiments, protein quantification is accomplished by comparing the intensities of reporter ions produced during MS/MS [11]. Since this approach enables sensitive and precise protein quantification, many research groups have successfully used TMT-based strategies [13–16].

With the aid of 10-plex TMT quantification strategy combined with high-resolution MS, we constructed a comprehensive proteome map of the newly developed mouse models. We have successfully discovered nearly 10,000 proteins and quantified 7000 proteins from the hippocampus of wild type, ADLP^APP/PS1^, ADLP^Tau^, and ADLP^APT^ mice. The protein abundances of ADLP^APT^ mice were compared with those of other single transgenic mice to discover differentially expressed proteins and characterize functional signatures of ADLP^APT^ mice via bioinformatics analysis. Furthermore, our network analysis could suggest the presence of interacting proteins that connect between amyloid and NFT pathologies. In conclusion, new ADLP^APT^ mice and their hippocampal proteome dataset may help to offer a novel insight of pathogenesis of AD in further studies targeting the concurrent molecular network of amyloid and NFT pathologies.

## Methods

### Experimental design

The aim of this study was to construct a mouse model of Alzheimer’s disease that carries mutant human genes and to introduce its molecular and functional characteristics. The protein expressions of newly constructed mouse models were assessed by quantitative proteomics combined with LC-MS/MS and TMT isobaric labeling. A total of 36 hippocampi samples were used in the proteomic experiments. (4 mouse types * 3 age-points * biological triplicates), which were randomly divided into four 10-plex TMT experimental sets. All samples were analyzed twice via MS. Three to twelve mice were sacrificed accordingly to the type of biochemical experiment, which includes western blotting and immunostaining. Behavioral tests of the AD model mice were performed by investigators in blind with respect to genotypes. No data were excluded.

### Reagents and materials

Tandem mass tag (TMT) 10-plex isobaric reagents, bicinchoninic acid (BCA) assay kit - reducing agent compatible, tris (2-carboxyethyl) phosphine (TCEP), and LC/MS-grade solvents such as acetone, acetonitrile (ACN), and water were purchased from Thermo Fisher Scientific (Waltham, MA). Other reagents and materials were purchased from the following companies: Dithiothreitol (DTT) and urea from AMRESCO (Solon, OH), Sodium dodecyl sulfate (SDS), Trizma base from USB (Cleveland, OH) and sequencing-grade modified trypsin from Promega Corporation (Madison, WI), POROS20 R2 bead from Applied Biosystems (Foster City, CA). High-purity (>97%) mass spectrometry (MS) grade ovalbumin from Protea (Morgantown, WV), HLB OASIS column from Waters (Milford, MA). All other reagents, unless noted, were purchased from Sigma-Aldrich (St. Louis, MO).

### Transgenic mice

5XFAD mice (Tg6799; Jackson Laboratory, Stock#006554) express both mutant human *APP* with the Swedish, Florida, and London mutations and mutant human *PSEN1* with the M146 L and L286 V mutations under the murine Thy1 promoter. JNPL3 mice (TauP301L-JNPL3; Taconic, Stock#2508 homozygote) carry mutant human tau with the P301L mutation under the murine prion protein promoter. Due to the mixed genetic background of JNPL3 mice, JNPL3 mice were backcrossed with B6SJL (C57BL/6 X SJL) mice. The resulting JNPL3 mice on the B6SJL genetic background were crossed with 5XFAD mice to create a novel animal model, ADLP animal model. This carries the three human mutant genes and its corresponding mutations mentioned before. Only female mice were used for pathological characterization due to earlier signs of aggravated pathologies and memory deficit than male mice.

### Immunohistochemistry (IHC)

Mice were anesthetized and perfused with 4% paraformaldehyde (PFA) solution in phosphate-buffered saline (PBS). The brain tissues were fixed with 4% PFA for 20 h at 4 °C, incubated in 30% sucrose (wt/vol) for 72 h and then frozen. The frozen brains were cut into 30 μm coronal sections using a Leica CM 1850 Cryostat. Brain slices were washed with PBS and then incubated in 70% formic acid in PBS for 20 min to perform antigen retrieval when amyloid plaques and NFTs were stained. Brain slices were permeabilized and blocked with blocking solution (0.3% Triton X-100, 5% horse serum, and 0.05% BSA solution) for 1 h at 20 °C prior to incubating it with primary antibodies overnight. Amyloid plaques were stained with the biotin-4G8 antibody (1:700, COVANCE), followed by the streptavidin-488-conjugated secondary antibody (Invitrogen). Hyperphosphorylated tau was examined by using the AT8 (1:300, Thermo Scientific) and AT180 (1:300, Thermo Scientific) antibody, which recognize the Ser202/Thr205 epitopes and Thr231 of human tau, followed by biotinylated anti-mouse IgG (Vector Laboratories) and streptavidin-594-conjugated secondary antibody (Invitrogen). To visualize astrocytes and microglia, anti-GFAP (1:1000, Invitrogen) for astrocytes and anti-Iba-1 (1:500, Wako) antibodies were used respectively. Hippocampal neurons of the CA1 layer were visualized by staining with anti-NeuN (1:1000, Millipore) antibody. Stained brain slices were incubated with goat anti-rat Alexa 488, donkey anti-rabbit Alexa 488, and donkey anti-mouse Alexa 647 antibody (1:500, Life Technologies) for 1 h at 20 °C. Images were obtained using LSM 700 (Carl Zeiss). At least six serial sections of each sample were imaged to consider the volume of cells in brain slices. When the number of neuronal cells in CA1 layer stained by anti-NeuN antibody were counted, one middle region of the hippocampus tissue was imaged to avoid an overlap of CA1 pyramidal neurons. All images were quantified using ImageJ software (NIH).

### Sarkosyl-insoluble tau fractionation

One side of the hippocampus was homogenized in 8 volumes of Tris buffer solution (TBS) including phosphatase inhibitors and protease by tissue grinder [TBS solution; 25 mM Tris/HCl, pH 7.4, 150 mM NaCl, 1 mM EDTA, 1 mM EGTA, phenylmethylsulfonyl fluoride (PMSF), protease 1, and 2]. The homogenates were centrifuged at 14,000 x g at 4 °C for 15 min. The supernatant was collected for further fractionation. The supernatant was incubated with N-Lauroylsarcosine sodium salt solution 20% solution (1% final concentration) at 37 °C rotator for 1 h and then ultracentrifuged at 150,000 x g at 25 °C for 1 h. The resulting sarkosyl-insoluble pellets were resuspended in TBS solution for washing pellets. The mixture was concentrated by ultracentrifuge again at 150,000 x g at 25 °C for 1 h. The resulting pellets containing tau aggregates were suspended with 5xSample buffer (Serva Blue G) and heated at 70 °C for 10 min to prevent further aggregation.

### Behavioral test

For Y-maze test, after introduction to the middle of the maze, the mouse was allowed to freely explore new environments for 8 min. Spatial memory function was measured as the percent of spontaneous alteration [17]. The number of total arm entries and the sequence of the Y-maze arm into which mice entered were recorded in order to calculate the percentage of spontaneous alteration. The number of alternations was counted when the mouse entered into the three different maze arms consecutively. The percent of spontaneous alteration was calculated as the number of alterations divided by the total entry number multiplied by 100.

### RT-PCR analysis

To verify the amount of mouse or human tau mRNA levels in ADLP^Tau^ and ADLP^APT^ mice, RT-PCR analysis was carried out with 10-month-old ADLP model mice. Total RNA was extracted from the hippocampus with the RNeasy Mini kit (QIAGEN). All RNA samples were converted into cDNA using Maxime RT PreMix Kit (iNtRON BIOTECH). Quantitative RT-PCR was carried out in triplicates using KAPA SYBR FAST ABI Prism qPCR kit (KAPA Biosystems). For mouse Tau, the primers 5’-AGCCCTAAGACTCCTCCA-3′ and 5’-TGCTGTAGCCGCTTCGTTCT-3′ were used. Human tau was amplified with the primers 5’-CTCCAAAATCAGGGGATCGC-3′ and 5’-CCTTGCTCAGGTC AACTGGT-3′. The mRNA levels of mouse Tau and human Tau were normalized with GAPDH which was amplified with the primers 5’-GGCCTTGACTGTGCCGTTGAATTT-3′ and 5’-ACAGCCGCATC TTCTTGTGCAGTG-3′. Once the reaction was completed, the RT-PCR products were evaluated/analyzed via gel-electrophoresis to measure/calculate the specificity of human tau primers.

### Western blot analysis

Western blot analysis was used to confirm tau aggregates from sarkosyl-insoluble tau fractionation and the validation of proteomic analysis results. After isolated tau aggregates were heated at 70 °C for 10 min to prevent further tau protein aggregation, the same volume of sarkosyl-insoluble tau aggregate samples was loaded per lane of 4–12% Bis-Tris polyacrylamide precast gels (NuPAGE system, Invitrogen). Following electrophoresis, proteins were transferred to a PVDF membrane. Membranes were blocked with 5% skim milk solution and then incubated with primary antibodies against human tau (Tau13, Abcam, 1:1000) and total tau (endogenous tau and human tau) (Tau5, Abcam, 1:1000). Primary antibodies against ABCA1 (Abcam, 1:500), Ptprc (CD45, Abcam, 1:1000) and Hcls1 (HS1, CST, 1:1000) were used for the validation of proteomic analysis. For confirmation of kinase expression levels, CK1δ (Abcam, 1:5000), RSK1 (CST, 1:2000) and GSK-3β (CST, 1:2000) were used as primary antibodies. Anti-mouse or rabbit IgG conjugated HRP was used to detect primary antibodies and West Save Gold (Ab frontier) was used for their visualization. Since sarkosyl-insoluble fractionation only extracts protein aggregates, certain proteins generally used for normalization such as GAPDH and β-actin were not detected in the sarkosyl-insoluble pellets. Thus, total antibody signals of tau aggregates or each signal for distinct sizes of tau aggregates were quantified for quantification.

### Mass spectrometry-based proteomics

Mouse hippocampus tissues were resected and subjected to the previously described sample preparation methods with some modifications [18–20]. Detailed procedures including protein digestion, peptide labeling, fractionation, and MS analysis are described in Additional file 1: Supplementary Methods.

### Quantification of protein abundance and statistics

The quantification and statistical processing methods described below are related to proteomic data. First of all, among the 9814 identified proteins, only 6964 proteins satisfying the following criteria were used for subsequent quantitative analysis; identification in all channels (7022 proteins), high protein confidence (6970 proteins, assessed by Proteome Discoverer), and possessing 1 or more unique peptides (6964 proteins). The protein abundance ratio of individual samples to pooled sample (named “normalized protein abundance”) was generated by dividing the reporter ion intensity of each channel by the intensity of the pooled sample channel in its corresponding experimental TMT set (Additional file 2: Figure S3B). There was no significant difference between the values of the pooled sample channels (Additional file 2: Figure S5E). Thus, the denominators were considered to be common and eliminated. The fold-change values used in the bioinformatics analysis were generated by dividing the normalized protein abundance of each transgenic mouse by the value of the age-matched wild type mouse. The distribution of ratiometric data was almost normal (Additional file 2: Figure S5G) but this was not thoroughly tested.

Statistical processes for the proteomic data were performed based on the normalized protein abundance using Perseus [21]. Initially, total identified proteins were filtered based on the 6964 proteins that quantified in all mouse samples. The statistical cut-off value for significance was set to *p*-value <0.05 for the Student’s t-test, while Benjamini-Hochberg FDR adjusted p-value cut-off [22] of 0.05 was applied for the ANOVA test. The normalized protein abundances were subjected to z-normalization followed by hierarchical clustering. The statistical tests for the other biochemical experiments were described in each figure legend.

### Bioinformatics analysis

The Gene Ontology (GO) of the proteins was classified using DAVID bioinformatics tool (version 6.8) [23]. The GO classification was evaluated by Fisher’s exact test to obtain a set of *P*-values, which were then filtered at a cut-off value of 0.01. Canonical pathways, downstream biological functions, and upstream regulators were enriched using Ingenuity Pathway Analysis (IPA, QIAGEN) [24]. The analytical algorithms embedded in IPA uses input protein list (here differentially expressed proteins) to predict putative upstream regulators such as transcription factors and growth factors, as well as downstream effects on known biological pathways. IPA derives these protein set-pathway (or regulator) relationships from their own large-scale causal network database, named Ingenuity Knowledge Base. Because the algorithm cannot determine with certainty which causalities in its database can explain our experimental results, the tool performs statistical tests (i.e. Fisher’s exact test) to assess the reliability of predicted upstream genes and pathways. Finally, IPA also assigns activation states (activated or inhibited) to putative regulators or pathways based on the quantitative values of protein members. The user will be given a confidence in the *P*-value obtained from the Fisher’s exact test and the magnitude of activation as a Z-score, respectively. In this study, the *P*-value cut-off criteria for the enrichment was 0.01 for Fig. 6a and b and the predictive activation Z-score cut-off was 1. For the Fig. 4b, the cut-off value was 0.05 and the predictive activation Z-score cut-off was 1. The initial pathway diagrams were obtained by IPA but were manually modified. Protein-protein interactions (PPIs) for the network analysis was interrogated from STRING database (http://www.string-db.org) [25]. The PPIs in network model were visualized using Cytoscape [26].

## Results

### Pathological symptoms of ADLP^APT^ mice

**Fig. 1:**
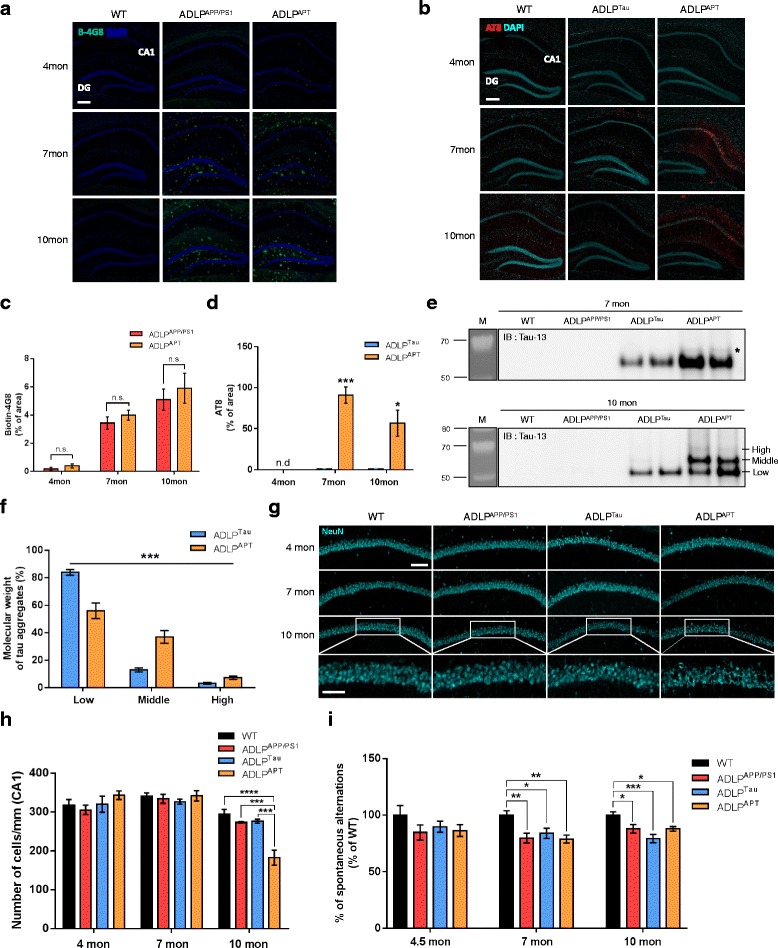
Pathological characterization of a novel animal model of Alzheimer’s disease. **a** and **c** For detection of amyloid plaques, hippocampal regions of ADLP mice were immunostained with the biotin-4G8 antibody at 4-, 7-, and 10-month-old. The percentage of amyloid plaques area was not significantly different between ADLP^APP/PS1^ and ADLP^APT^ mice (Student’s t-test, *n* = 3–4 per group). Scale bar represents 200 μm. **b** and **d**) The hippocampal CA1 layer of ADLP mice was stained with the AT8 antibody against phosphorylated tau (Ser202/Thr205). A significant increase in AT8 immunoreactivity was observed in ADLP^APT^ mice compared with age-matched ADLP^Tau^ mice (Student’s t-test, *n* = 3–4 per group). Scale bar represents 200 μm. **e** Sarkosyl-insoluble tau fractions from 7 and 10 months ADLP mice hippocampus were analyzed by western blot analysis using human tau specific antibody (Tau13). **f** Each distinct size of sarkosyl-insoluble tau was quantified in 10 months old ADLP^Tau^ and ADLP^APT^ mice (Chi-square test; *n* = 6 mice per genotypes). **g** The CA1 pyramidal neurons of ADLP mice were stained with anti-NeuN antibody to determine degrees of neuronal loss. Scale bars represents 100 μm or 50 μm (enlarged figures). **h** Quantification of the number of CA1 neurons in 7- and 10-month-old ADLP mouse model (one-way ANOVA in each age of ADLP mouse model). **i** ADLP model mice showed memory impairment compared with wild-type mice, which examined by the Y-maze test (one-way ANOVA in each age of ADLP mouse model, *n* = 9–11 per group). Results are expressed as mean ± SEM. * *P* < 0.05, ** *P* < 0.01, *** *P* < 0.001, **** *P* < 0.0001

Four mouse models with the same genetic background were generated: wild-type, ADLP^APP/PS1^ (Aβ accumulation only), ADLP^Tau^ (NFTs only), and ADLP^APT^ (both Aβ and NFTs) mice. To confirm the amyloid plaque burden in the hippocampus of the ADLP mice, we stained coronal brain sections of 4-, 7-, and 10-month-old ADLP mice with the biotin-4G8 antibody that recognizes amino acids 17–24 of Aβ peptides. Both ADLP^APP/PS1^ and ADLP^APT^ mice started showing slight extracellular accumulation of Aβ at 4 months and exhibited large amyloid plaques at 7 months; no significance was observed between-model difference in their amyloid plaque burdens (Fig. 1a and c).

To investigate the NFT pathology in the hippocampus, we first stained phosphorylated tau using the AT8 antibody, which detects residue Ser202/Thr205 of phosphorylated tau in paired helical filaments (PHFs). Intense immunoreactivity of PHFs against phosphorylated tau was observed in the hippocampal CA1 area of 7- and 10-month-old ADLP^APT^ mice compared with ADLP^Tau^ mice (Fig. 1b and d). Subsequently, we used the AT180 antibody to detect the residue Thr231 of phosphorylated tau. The residue Thr231 was strongly stained at the soma and dendrites of CA1 neurons starting from 4 months of age. In 10-month-old mice, we observed phosphorylated tau localized only at the soma region as a form of NFTs, indicating that ADLP^APT^ mice developed severe NFT pathology compared with ADLP^Tau^ mice (Additional file 2: Figure S1A).

To validate the expression of tau in the hippocampus, mRNA levels of endogenous mouse tau were confirmed by RT-PCR analysis. The results indicated no significant differences among the ADLP mouse models. Furthermore, the human tau mRNA expression was not different between ADLP^Tau^ and ADLP^APT^ mice, indicating accelerated NFT pathology in ADLP^APT^ mice without quantitative difference in tau protein (Additional file 2: Figure S1B–1D). Subsequently, we isolated NFTs from the hippocampus of 7- and 10-month-old ADLP^Tau^ and ADLP^APT^ mice using sarkosyl-insoluble tau fractionation. Tau aggregates in ADLP^APT^ mice were composed of higher molecular weight of tau at both 7 and 10 months of age, and they appeared at regular intervals above the 55 kDa of human tau. The proportion of the higher molecular weight tau increased in 10-month-old ADLP^APT^ mice as compared to age-matched ADLP^Tau^ mice (Fig. 1e). However, ADLP^Tau^ mice had only low-molecular-weight tau as a main constituent until 10 months of age (Fig. 1e and f). This suggests that the phosphorylation of human tau was increased by the amyloid pathology in ADLP^APT^ mice. Using the Tau5 antibody, which reacts to both mouse and human tau, we confirmed that sarkosyl-insoluble tau fractionation was the appropriate method for specifically extracting tau aggregates from the hippocampus of ADLP^Tau^ and ADLP^APT^ mice (Additional file 2: Figure S1E).

We also examined whether the accelerated NFT pathology observed in the hippocampal CA1 area of ADLP^APT^ mice was associated with neuronal loss in the CA1 layer. Neuronal quantification using NeuN immunostaining revealed a significant decrease in the number of CA1 neurons in 10-month-old ADLP^APT^ mice, but not in the other models, indicating that the amyloid pathology of this model aggravated neuronal death via the NFT pathology (Fig. 1g and h). Because spatial memory impairment is an essential feature in AD animal models [27], we used the alternation Y-maze task to evaluate spatial memory in ADLP mice at different ages. Although there was no memory deficit in 4-month-old ADLP^APT^ mice, they showed significant deficits in spatial memory beginning at 7 months (Fig. 1i). Altogether, these data indicated that ADLP^APT^ mice exhibit an accelerated NFT pathology, amyloid pathology, neuronal loss, and age-related memory impairment. Thus, this model appears to comprehensively reflect the pathophysiological changes in AD.

### Constructing a Hippocampal proteome database of the ADLP mouse models

**Fig. 2:**
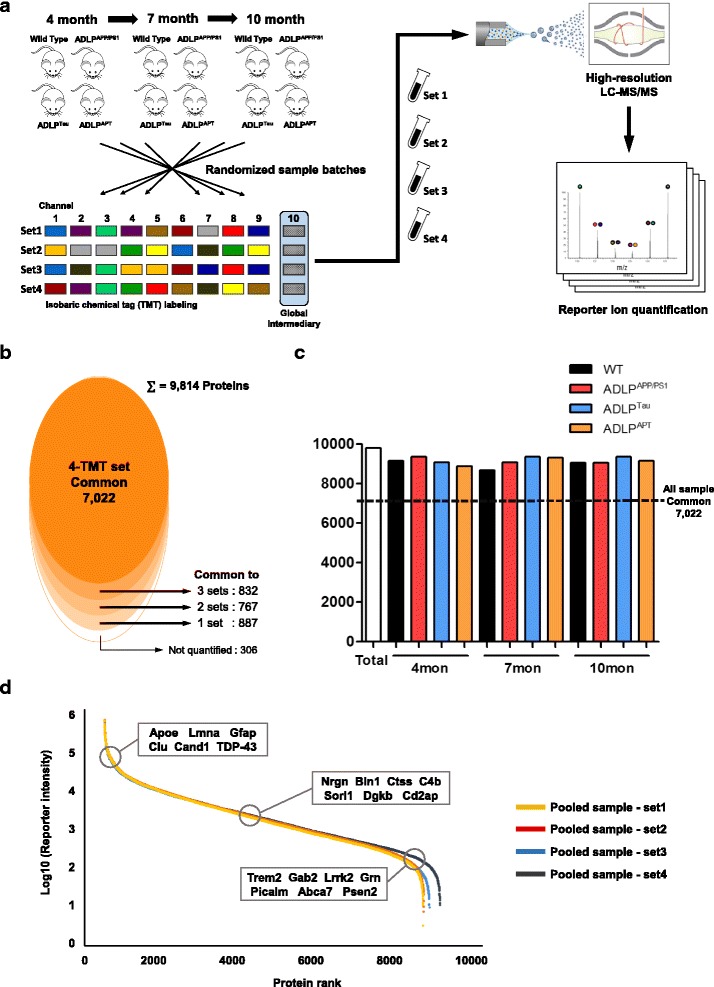
Hippocampal proteome analysis of ADLP model mice. **a** Graphical illustration of the workflow used for our TMT-based proteomic analysis. The detailed sample assignments for TMT labeling are shown in Fig. S2A. **b** A total of 9814 protein groups were identified in our study; of them, 7022 protein groups were identified and quantified in all experimental sets. The “Not quantified” proteins were identified via search algorithm, but their reporter ions were not detected and were excluded from subsequent quantitative analysis. **c** A bar chart showing the number of proteins identified in the hippocampus of each ADLP model. **d** Dynamic range of protein abundance, spanning six orders of magnitude. Normalized reporter ion intensities of pooled samples in each experimental set were used. Proteins known as AD risk factors are annotated in the plot

To obtain insights into the molecular basis of AD pathogenesis, we performed quantitative proteomic analysis using three replicates of hippocampus resected from each mouse model at three time points (4, 7, and 10 months of age; Fig. 2a). Briefly, each hippocampus was homogenized and digested via filter-associated sample preparation [28], and the peptide samples were labeled with TMT reagents. The labeled samples were analyzed using Q Exactive mass spectrometer. The resulting 96 raw files (12 peptide fractions × 4 TMT-mix experimental sets × technical duplicates) were processed in Proteome Discoverer based on the SEQUEST-HT algorithm. In total, 9814 protein groups were identified from 125,683 unique peptides; among them, 7022 protein groups were successfully identified in all 36 samples (Fig. 2b and c). The expression levels of the mutated human proteins (APP, PSEN1, and tau) were clearly verified by our MS analysis (Additional file 2: Figure S1F–H). Our proteomic platform allowed us to discover in-depth proteome which dynamic range covered over six orders of magnitude. This comprehensive dataset included a number of well-known risk factors of AD (http://www.alzgene.org), such as *Apoe* [29], *Clu* [30], and TDP-43 (*Tardbp*) [31, 32], which were quantified in all samples. Notably, we also detected low-abundance risk factors, such as *Gab2* [33–35], *Trem2* [36], and *Abca7* [37], indicating that our proteome exhibited a profound quantification depth (Fig. 2d). The other genes shown in Fig. 2d were reported as AD risk factors in other studies [38–47]. Thus, our extensive proteome profiling of the mouse hippocampus yielded a database that could form a solid foundation for further biological interpretations. All protein identification data are presented in Additional file 3: Table S1. The proteins quantified in all 36 channels are listed in Additional file 4: Table S2. In addition, we have provided the interactive plot that shows the protein changes over three age points for the input gene in “Profile plot” sheet of Additional file 4: Table S2 [48].

To assess the comprehensiveness of the proteome data, we compared our data with those in a recently reported mouse brain proteome atlas (http://www.mousebrainproteome.com) [9]. The comparative analysis showed that approximately 87% of the protein groups in this dataset, which is the largest to date, were also identified in our study. Notably, despite using less instrument time than the label-free method used in constructing the mouse brain atlas, our TMT-based proteomics technique has constructed a proteome dataset of comparable depth. Furthermore, 2344 genes were uniquely identified in our dataset, indicating that our data expanded the hippocampal proteome to a great extent using a single type of sample on a more time-efficient platform (Additional file 2: Figure S4A). In addition, we compared the efficiency of protein identification with those reported in other proteomic studies of transgenic AD mouse models; our dataset contained the highest number of protein identifications (Additional file 2: Figure S4B).

### Quality assessment of the proteomic data

The multiplexing capacities of the TMT-based workflow allowed us to characterize the quantitative variations within and between hippocampal samples. Our analysis of a non-homologous (i.e., ovalbumin) spiked-in standard for inter- and intrabatch normalization revealed a coefficient of variation of 6.3% during repetitive MS analysis (Additional file 2: Figure S5A). Although the intensity variations among non-normalized biological replicates showed a suitable reproducibility, we observed a slight improvement of reproducibility when the normalization was performed using ovalbumin (Additional file 2: Figure S5B and C). Thus, the reporter ion intensities of proteins were initially normalized with respect to the intensity of ovalbumin.

To investigate the quantitative reproducibility (technical and experimental set-to-set variations), we used protein abundances in pooled sample channels (131) to perform cross-correlation examinations. Based on the results, the duplicate MS analysis showed excellent consistency, with an average R^2^ correlation value of 0.994 (Additional file 2: Figure S5D). The mean R^2^ value of the quantified protein abundance between the TMT experimental sets was excellent at 0.992 (range, 0.988–0.995) (Additional file 2: Figure S5E). Furthermore, the results indicated good correlation among the biological replicates, with a mean Pearson’s correlation value of 0.991 (Additional file 2: Figure S5F). Thus, the observed differences in protein expression reflected the molecular diversity among the mouse histotypes, not the effect of our TMT-based strategy [49].

### Proteome alterations in ADLP hippocampus

**Fig. 3:**
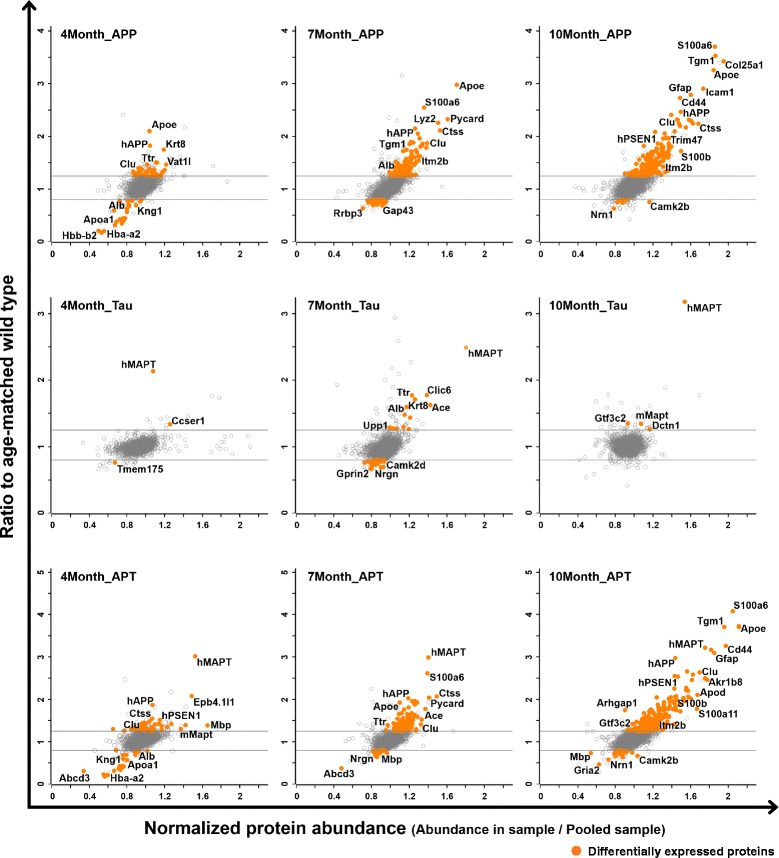
Abundant and enriched proteins in the hippocampus of transgenic ADLP model mice. Scatter plot of relative protein expression levels in ADLP versus wild-type mice (y-axis). The x-axis represents the normalized protein abundance (ratio to pooled sample). Each graph was vertically placed along with ADLP model type and horizontally placed in order of age/disease progression. Significantly regulated proteins (Student’s T-test *p*-value <0.05 and fold-change cut-off: 1.25) are shown as orange circles. *APP = ADLP^APP/PS1^, Tau = ADLP^Tau^, APT = ADLP^APT^ mice

To resolve the regulated proteins in each ADLP mouse model, we quantitatively analyzed the protein expression in a ratiometric manner (see Methods). Based on the fold-change in the normalized protein abundance, we identified 63, 178, and 245 proteins that were significantly regulated in 4-, 7-, and 10-month-old ADLP^APP/PS1^ mice, respectively (Student’s *t*-test, *p*-value <0.05, fold-change >1.25). In comparison, 3, 42, and 4 proteins were significantly regulated in 4-, 7-, and 10-month-old ADLP^Tau^ mice, respectively, whereas 62, 131, and 311 proteins were significantly regulated in 4-, 7-, and 10-month-old ADLP^APT^ mice, respectively. The scatter plots showed that more proteins were up-regulated during the progression of amyloid pathology than during that of NFT pathology. Interestingly, the overall tendencies of the significantly regulated proteins were similar in ADLP^APP/PS1^ and ADLP^APT^ mice, indicating that the amyloid pathology exerted stronger effects on the overall proteome than did the NFT pathology (Fig. 3). The proteins with significant change in each model are summarized in Additional file 5: Table S3.

**Fig. 4:**
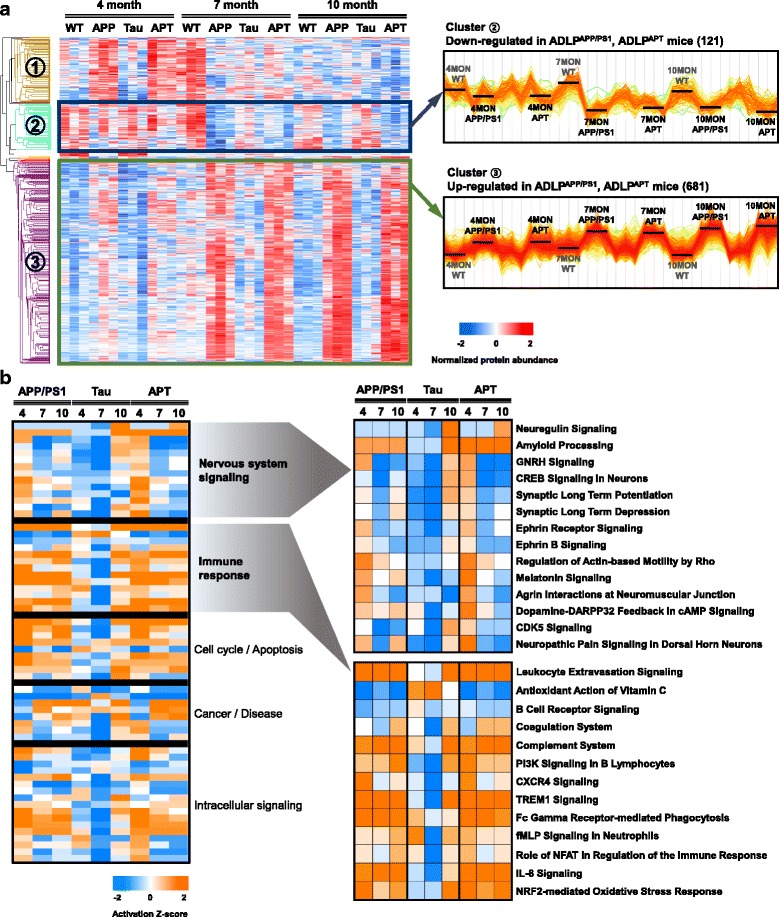
Protein quantitation overview and comparative pathway enrichment analysis of ADLP model mice. **a** Hierarchical clustering of differentially expressed proteins (DEPs) across different transgenic types and ages (ANOVA FDR < 0.05). Protein expression profiles were largely clustered into three patterns; The decreasing pattern (Cluster 2) and the increasing pattern (Cluster 3) of the mouse models with human *APP* (ADLP ^APP/PS1^, ADLP ^APT^) are prominent. The right panel shows the Z-normalized protein abundance according to the mouse samples as profile plots. **b** Canonical pathway enrichment for DEPs. The categories of nervous system signaling and immune response were overrepresented (~50%) and are highlighted. The significant pathways (Fisher’s exact test *p*-value <0.05) were deduced using Ingenuity Pathway Analysis (IPA) and their predictive activation/inhibition status is represented as the Z-score. *APP/PS1 = ADLP^APP/PS1^, Tau = ADLP^Tau^, APT = ADLP^APT^ mice

To identify the specific protein changes under the various AD pathologies (Aβ, Tau, or both Aβ and Tau), we used ANOVA to investigate the global diversity. Our ANOVA indicated that 1094 protein groups were differentially expressed proteins (DEPs) among the ADLP mouse models (FDR cut-off <0.05; Additional file 6: Table S4). Hierarchical clustering divided these proteins into three groups, including up-regulated proteins (cluster 2; 681 proteins) and down-regulated proteins (cluster 3; 121 proteins). ADLP^Tau^ mice had relatively few DEPs, whereas ADLP^APP/PS1^ and ADLP^APT^ mice shared DEPs that showed drastic age-related perturbations (Fig. 4a). This suggests that tau itself is insufficient to drive the hippocampal proteome regulation during the stage up to 10 months of AD pathogenesis.

We subsequently investigated how these DEPs affected downstream pathways in an unbiased manner. The ingenuity pathway analysis (IPA) platform identified five canonical pathway categories (nervous system signaling, immune response, cell cycle/apoptosis, cancer/disease signaling, and intracellular signaling pathways) that were significantly enriched by the DEPs (*p*-value <0.05; activation Z-score > 1; Fig. 4b). The primary enriched pathway categories were nervous system signaling and the immune response, which are known to be associated with AD pathogenesis. While the signaling pathways of the nervous system tend to become inactive with age, most identified immune response pathways were predicted to be activated as the disease progressed. The exact Z-scores and *p*-values for the IPA results are listed in Additional file 7: Table S5.

### Identifying molecular and functional signatures associated with pathology of ADLP^APT^ mice

**Fig. 5:**
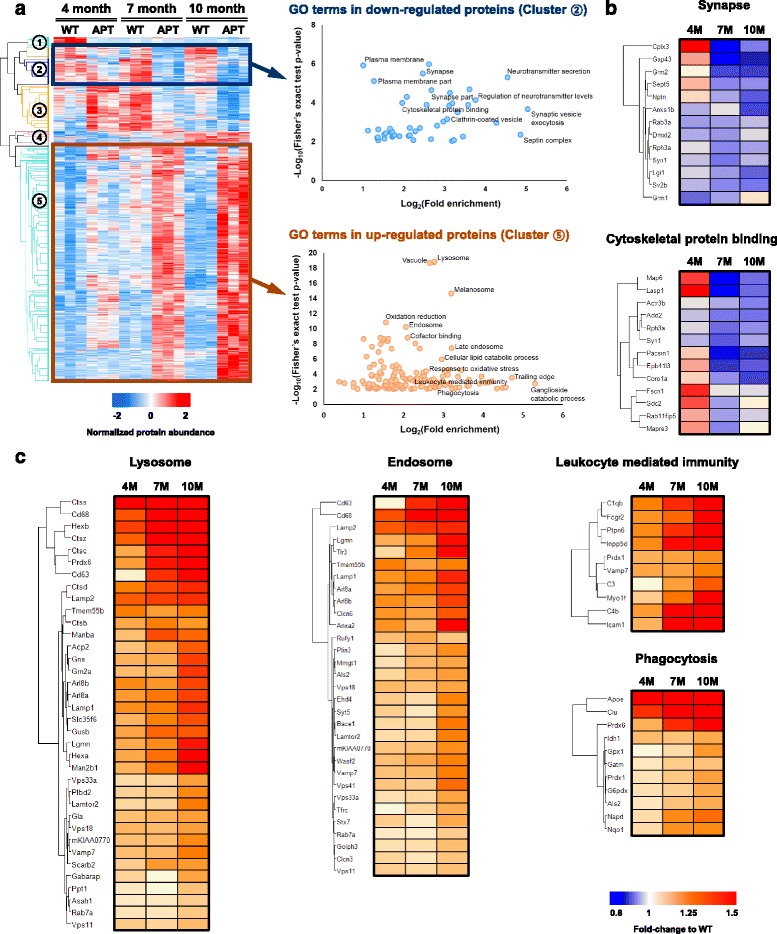
Protein clustering reveals major biological and molecular signatures of ADLP^APT^ mice. **a** Hierarchical clustering of differentially expressed proteins (DEP^APT^s) between ADLP^APT^ and wild-type mice (732 proteins; ANOVA FDR < 0.05). The DEPs^APT^ were clustered into five types of expression pattern. The protein expression level is the normalized protein abundance represented after Z-normalization. Scatter plot of enriched Gene Ontology (GO) terms in protein cluster 2 (down-regulated proteins) and cluster 5 (up-regulated proteins) are shown in the right panel. The -log10 (*P*-value) is plotted against the fold-enrichment of each GO term. **b** Heatmaps showing the expression levels of the proteins involved in the GO terms, such as synapse proteins and cytoskeleton binding proteins, in cluster 2 (down-regulated proteins). **c** Heatmaps for the expression levels of co-regulated proteins corresponding to the GO terms, such as lysosome, endosome, leukocyte-mediated immunity, and phagocytosis, in cluster 5 (up-regulated proteins)

We next investigated the age-dependent dysregulation of biological functions in ADLP^APT^ mice. We identified 732 proteins that were differentially expressed with respect to ADLP^WT^ mice (DEPs^APT^, ANOVA FDR cut-off <0.05; Additional file 8: Table S6). Our hierarchical clustering analysis divided the DEPs^APT^ into five clusters, including gradually down-regulated proteins (cluster 2; 79 proteins) and gradually up-regulated proteins (cluster 5; 499 proteins; Fig. 5a). Gene ontology (GO) enrichment analysis revealed that the proteins in cluster 2 were particularly involved in synapses and cytoskeleton binding, suggesting that the AD pathologies gradually damage the synaptic plasticity in the hippocampus of ADLP^APT^ mice (Fig. 5a, right panel, and Fig 5b). The proteins of cluster 5 were primarily assigned to the inflammatory and degradation systems, such as leukocyte-mediated immunity, phagocytosis, endosomes, and lysosomes, indicating that AD pathologies could activate both inflammation and degradation systems to remove Aβ plaques and NFTs (Fig. 5c and Additional file 9: Table S7).

**Fig. 6:**
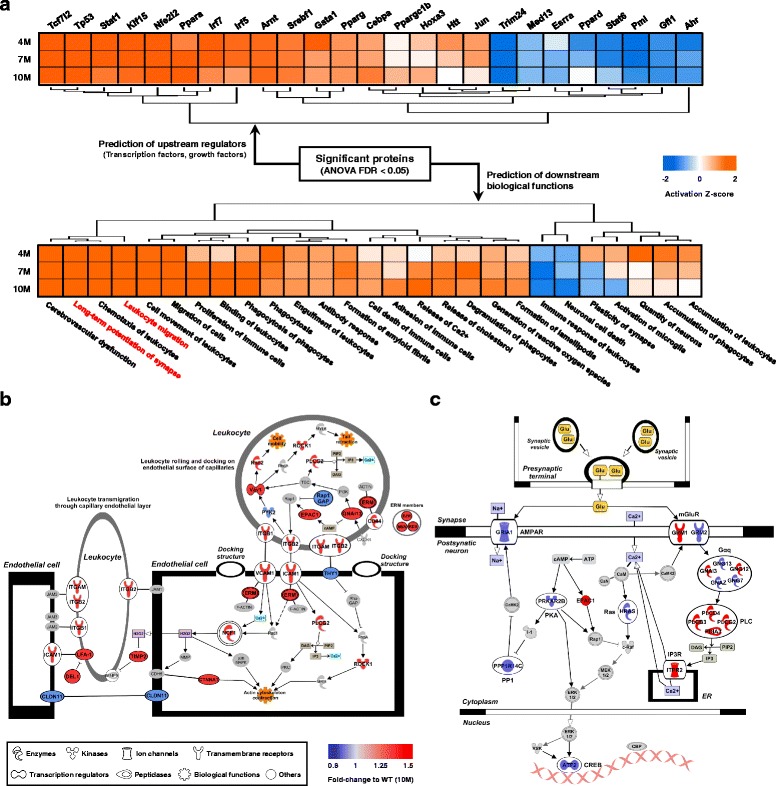
Prediction of molecular responses to AD pathology in ADLP^APT^ mice. **a** Hierarchical clustering of downstream biological functions (lower panel) and the upstream regulators (upper panel) assessed by IPA using 732 DEPs^APT^ (ANOVA FDR < 0.05). The predictive activation/inhibition status is shown as a Z-score. **b** Graphical representation of the protein expression levels of members of the leukocyte extravasation pathway and (**c**) the synaptic long-term potentiation pathway in 10-month-old ADLP^APT^ mice. Individual proteins’ fold-change values to the wild type were visualized in color (blue: down-regulation, red: up-regulation). Glu = Glutamate, PIP2 = Phosphatidylinositol 4,5-bisphosphate, DAG = Diacylglycerol, IP3 = Inositol 1,4,5-trisphosphate, ATP = Adenosine triphosphate, cAMP = Cyclic adenosine monophosphate

To define biological activities that contribute to the pathological phenotypes of ADLP^APT^ mice, we performed bioinformatics analysis using IPA to identify upstream regulators as well as downstream functions of DEPs^APT^ (Additional file 10: Table S8). The IPA predicted 25 relevant upstream regulators to modulate DEPs^APT^. In addition, DEPs^APT^ were assigned to 27 downstream biological functions associated with the immune response and the nervous system (p-value <0.01; Fig. 6a). To further explore the predictive biological pathways significantly altered in ADLP^APT^ mice, we investigated relevant canonical pathways (p-value <0.01; Additional file 10: Table S8). Among these, one of the most dysregulated immune response-related pathways was the leukocyte extravasation signaling pathway. Adhesion-related proteins, which are mainly expressed in peripheral immune cells and endothelial cells, were differentially expressed in 10-month-old ADLP^APT^ mice (Fig. 6b). We speculate that changes in endothelial cells are likely to reflect the AD pathology in the brain parenchyma and that such changes recruit peripheral immune cells into the brain parenchyma. Among the canonical pathways related to the nervous system, synaptic long-term potentiation (LTP) was a representative pathway altered in ADLP^APT^ mice (Fig. 6c). Post-synaptic proteins of this pathway were mainly dysregulated by the extracellular amyloid pathology and the intracellular NFT pathology. Thus, the biological pathway alterations seen in ADLP^APT^ mice are consistent with the pathological changes seen in the nervous system of patients with AD.

### Network analysis of unique proteome changes in ADLP^APT^ mice

**Fig. 7:**
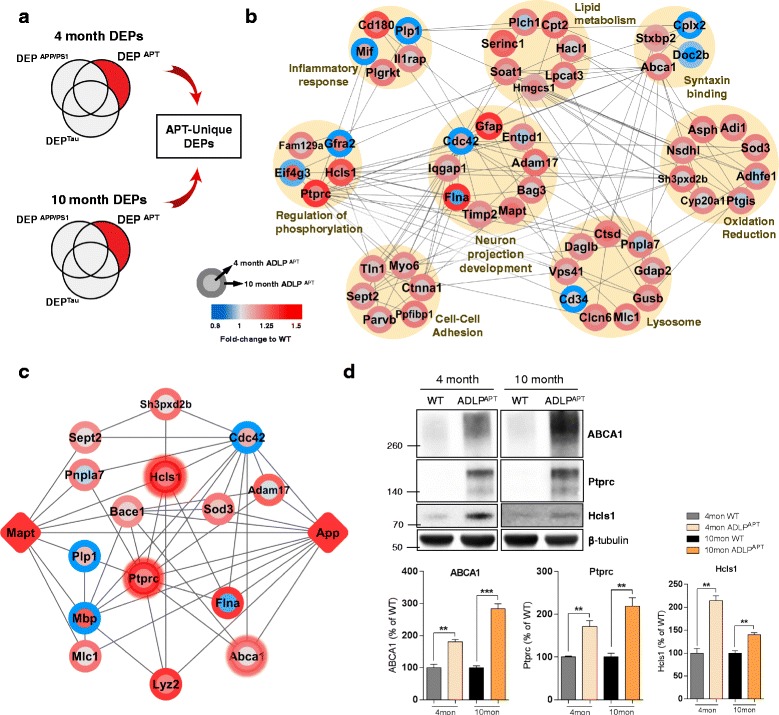
Exclusive DEPs in ADLP^APT^ mice and their related functional networks. **a** Proteins that were significantly altered in early or late ADLP^APT^ mice (Student’s t-test *p*-value <0.05 and fold-change >1.25) but not in the other models (fold change <1.25) were selected as exclusive DEPs^APT^. **b** The biological functions derived from the GO analysis are shown along with their networking with associated proteins. **c** The knowledge database-derived protein network of *App*, *Mapt*, and exclusive DEPs^APT^. The fold-change values of individual protein nodes were visualized in color circles (blue: down-regulation, red: up-regulation). The color of the inner circle is the fold-change of the 4-month ADLP^APT^ mouse, and the color of the outer circle represents that of the 10-month ADLP^APT^ mouse. **d** Three proteins that form bridge between *App* and *Mapt* (*Hcls1*, *Ptprc* and *Abca1*) were validated by western blot analysis. Representative western blot images are shown herein. The expression levels of the proteins are quantified compared with wild type mice (*n* = 3 per genotype for 4 and 10 months; Student’s t test). Results are expressed as the mean ± SEM. **P < 0.01; ***P < 0.001

To identify ADLP^APT^ mouse-specific protein changes, we investigated the exclusive DEPs^APT^ in 4- (early) and 10-month-old (late) ADLP^APT^ mice (Fig. 7a and Additional file 11: Table S9). Using the STRING database, we organized a protein–protein interaction map of the exclusive DEPs^APT^ representing expression changes by age and the GO categories into which these proteins could be classified. Our GO analysis revealed that the exclusive DEPs^APT^ were classified in the categories related to the immune response (e.g., cell–cell adhesion and inflammatory responses) and neuronal functions (e.g., syntaxin binding, lysosomes, and neuron projection development) (Fig. 7b). Subsequently, we examined the correlation of exclusive DEPs^APT^ with 2 AD-causative molecules, *App* and *Mapt*, in an effort to depict how the amyloid and NFT pathologies interconnect with one another. The constructed *App*–*Mapt* network showed the direct or indirect connections of proteins with *App* and *Mapt*, suggesting that these molecular targets could influence specific pathologies of ADLP^APT^ mice (Fig. 7c). The expression of *Abca1*, *Ptprc*, and *Hcls1* that form a protein–protein interaction bridge between *App* and *Mapt* were further validated by the western blot analysis (Fig. 7d). The expression levels of exclusive DEPs^APT^ in the other transgenic mice are presented in Additional file 2: Figure S6.

### Comparison of ADLP^APT^ mouse proteome with human AD proteome

We ultimately aimed to construct a mouse model that fully recapitulates the molecular and functional features of AD. To investigate how the ADLP^APT^ mouse model reflects AD, we performed a comparative analysis with the proteome data of a recently published human AD study [50]. This group performed proteomic analysis using brain tissues from patients with AD, elucidating molecular features and protein networks. Our dataset encompassed approximately 92% of the proteins identified in the human AD proteomics study. The expression patterns of DEPs common to both studies (Student’s *t*-test *p*-value <0.05) overlapped by 41 and 25% in up-regulated and down-regulated proteins, respectively (Additional file 2: Figure S4C). Although the numerical correlation between the two data was low (*R*^*2*^ < 0.3), the commonly significant proteins have been classified into functional categories related to AD. Particularly, 10 up-regulated proteins, such as *Clu*, *Mao-b*, *Cyp46a1*, and *Vps26a*, were deduced to have significant associations with AD (Additional file 12: Table S10). These results implicated that our mouse model adequately reflects the known molecular characteristics of AD and can thus be further utilized in the researches to discover new drug targets. Furthermore, the other proteins in the categories related to neurodegeneration may play a significant role in future studies. The results of the comparative analysis with human proteomic data are listed in Additional file 4: Table S2.

## Discussion

The amyloid cascade hypothesis claims that Aβ is the initial trigger for further pathological changes, including tau hyper-phosphorylation and NFT formation, which accelerate AD progression [51]. To investigate the prominent role of Aβ in causing AD pathogenesis, we developed ADLP^APT^ mice, a novel animal model of AD that develops more robust amyloid and NFT pathologies than do other available animal models. This model showed no aggravation of the amyloid pathology beyond that seen in ADLP^APP/PS1^ mice, indicating that the development of the NFT pathology does not affect the amyloid pathology. However, based on sarkosyl-insoluble tau fractionation for the aggregated form of tau and immunostaining for phosphorylated tau, the accelerated NFT pathology appeared in the hippocampus of ADLP^APT^ mice starting from 7 months of age in the absence of any observable difference in the transcription/expression of the human *MAPT* gene. Abnormal tau phosphorylation is a crucial event that triggers tau aggregation in AD brains [52]. Various kinases have been suggested to be involved in tau phosphorylation process. Among external stimuli to activate kinases, the role of Aβ for activating tau kinases has been reported in a downstream of Aβ toxicity [53]. In order to investigate whether increased kinase expression levels are causative factors for accelerating NFT pathology, we have confirmed the expression levels of three kinases (*Csnk1d*, *Gsk3-beta* and *Rps6ka1*) involved in tau phosphorylation by western blot analysis [54–56]. Notably, no differences were observed in the kinase levels between wild-type and ADLP^APT^ mice with the disease progression. In addition to western blot analysis, the MS results also indicated no changes in kinase expression levels. As for the kinases not checked by western blot analysis (e.g. *Cdk5*, *Src*, *Fyn* and *Abl1*), they were not changed among all groups, as confirmed by our hippocampal proteome data. These results support that accelerated NFT pathology in ADLP^APT^ mice hippocampus is not accompanied by changes of tau kinases (Additional file 2: Figure S7). Previous studies have hypothesized that Aβ is the initiator of the NFT pathology [53, 57, 58]. Indeed, Aβ affects tau kinase activity or localization resulting in tau toxicity in neurons. Moreover, the amyloid pathology in the cortex accelerates tau propagation process throughout the entorhinal cortex and aggravates the NFT pathology [57]. Because the underlying molecular mechanisms of the pathophysiological interaction between Aβ and tau are unclear, ADLP^APT^ mice may enable further exploration of the Aβ-tau axis hypothesis on the progression of AD pathogenesis.

To further investigate the biological functions altered in the hippocampus of ADLP^APT^ mice, we first constructed a hippocampal proteomic database of ADLP animal models using LC-MS. Notably, as the disease progressed, the protein expression profiles of ADLP^Tau^ mice resembled those of wild type mice, indicating that the relatively slow NFT pathology had a small impact on the proteomic changes during the early stage of the disease. In contrast, ADLP^APT^ mice showed dramatic proteome-wide changes with a pattern similar to that of ADLP^APP/PS1^ mice. One of the main explanations for the high similarity between ADLP^APP/PS1^ and ADLP^APT^ mice is the presence of inflammatory responses in the hippocampus. Almost all immune-related pathways exhibited similar changes over time in ADLP^APP/PS1^ and ADLP^APT^ mice, indicating that Aβ is one of the main factors for active inflammatory responses in the hippocampus (Fig. 4b). In both mice, the immune response categories exhibited changes at 4 months of age, even before the first deposition of amyloid plaques in the hippocampus. Immunohistochemical analysis confirmed similarities in the inflammation status between ADLP^APP/PS1^ and ADLP^APT^ mice, as evidenced by the staining with astrocytes and microglia markers in the hippocampus (Additional file 2: Figure S2). These findings indicate that the immune responses in the hippocampus were mainly influenced by the amyloid pathology regardless of the NFT pathology.

Moreover, GO analysis of the gradually up-regulated DEPs in ADLP^APT^ mice identified many glial cell type-related categories, such as phagocytosis, cell chemotaxis, and macrophage infiltration (cluster 5, Fig. 5a). One of the main dysregulated pathways in ADLP^APT^ mice was the leukocyte extravasation signaling pathway. Various adhesion-related molecules (*Icam-1*, *Vcam-1*, *Itgb-1/2*, and *Cd44*) were up-regulated in the hippocampus of ADLP^APT^ mice (Fig. 6a and b), indicating that AD pathologies can modulate endothelial cells in the brain parenchyma and recruit peripheral immune [59]. Both amyloid plaques and neurofibrillary tangles can activate inflammatory responses, thereby inducing the secretion of cytokines from microglia and astrocytes. Moreover, these serial pathologies also affect endothelial cells and disrupt the blood–brain barrier (BBB) integrity in AD [60]. A disrupted BBB with increased inflammatory cytokines finally results in the recruitment of peripheral immune cells toward brain parenchyma in AD. Several previous reports have suggested communication between peripheral immune cells and brain parenchyma as a pathological phenotype of AD, even if their functional roles remain unclear. Laurent C et al. have reported that CD8-positive T cells infiltrate the hippocampus. They have also demonstrated that the infiltration of CD8-positive lymphocytes in the cortical region of patients with frontotemporal dementia who develop tauopathy resulted from P301L mutation within the *tau* gene [61]. Previously, using two-photon microscopy, we have observed that neutrophils can extravasate from blood vessels into the brain parenchyma where amyloid plaques are present. In the brain parenchyma, the neutrophils adhere to amyloid plaques and phagocyte them [62]. Moreover, Zenaro E et al. have reported that the number of neutrophils adhere both inside blood vessels and in the brain parenchyma of patients with AD [63]. Depleting temporarily neutrophils at the early stage of disease progression in a transgenic mouse model of AD prevented memory impairments, suggesting that the infiltration of neutrophils contributes to the pathogenesis of AD. The up-regulation of the proteins involved in the leukocyte extravasation signaling in ADLP^APT^ mice suggested that peripheral immune cells may interact with capillary endothelial cells for infiltration into the brain parenchyma.

When glial or peripheral immune cells detect extracellular amyloid plaques, they clear the amyloid deposits through phagocytosis [64]. In addition to extracellular amyloid plaques, when neurons host NFTs in the cytosol, they activate the degradation system including the autophagy-lysosome system to eliminate them [65, 66]. Consistent with these, the GO analysis revealed that the gradually upregulated DEPs^APT^ included many components of the degradation system (cluster 5, Fig. 5c). In addition, constituents of the endosome GO category were upregulated, demonstrating that the endocytic pathway via endocytosis was also activated in ADLP^APT^ mice. Thus, although we could not clarify which cell types exhibited these protein changes, the results of the GO analysis of upregulated proteins collectively suggested that AD pathologies trigger a sequential response that moves from phagocytosis to degradation via the endocytic pathway.

Several nervous system signaling pathways appear to differ between ADLP^APP/PS1^ and ADLP^APT^ mice. The two canonical pathways, cAMP response element-binding protein (CREB) signaling in neurons and synaptic LTP, were inactivated only in the 10-month-old ADLP^APT^ mice (Fig. 6c). It is established that CREB signaling contributes to cognitive functions by modulating synaptic plasticity [67]. Proteins associated with LTP processes are increased by CREB signaling in the hippocampus; in AD, alterations in Ca^2+^ signaling lead to decreased CREB signaling and altered LTP [68]. Similarly, our ADLP^APT^ mice showed downregulated CREB signaling and LTP compared with ADLP^Tau^ and ADLP^APP/PS1^ mice. Thus, these two pathways are predicted to be disturbed simultaneously by both Aβ accumulation and NFTs. This suggests that ADLP^APT^ mice have pathological symptoms similar to those of patients with AD [69].

To investigate the putative mechanisms underlying the molecular pathogenesis of ADLP^APT^ mice, we sorted exclusive DEPs in ADLP^APT^ to identify unique molecular alterations. The protein–protein interaction map of exclusive DEPs^APT^ showed their direct or indirect interactions with each other and categories that each protein belongs to. We further investigated the molecular interactions of exclusive DEPs^APT^ with *App* and *Mapt* (Fig. 7c). The generated *App*–*Mapt* network contained 15 components that have been reported to interact among each other, although little is known about their relevance to the Aβ-tau axis. For example, *Ptprc* is a well-known microglia marker that has a strong association with Aβ oligomerization [70]. However, to our knowledge, the regulation of this protein in the hippocampus of AD mouse models is not well studied. In contrast, *Abca1*, a risk factor for AD, has been reported to play a significant role in Aβ clearance [71]. In addition, *Hcls1* is a leukocyte-specific actin-binding protein involved in immune response mechanisms [72]. We found that these proteins are involved in immune responses in the hippocampus, forming an interaction bridge on our *App*-*Mapt* network. Furthermore, western blot analysis clearly confirmed the regulation of these proteins in ADLP^APT^ mice. Since our *App*-*Mapt* protein network provides information on DEPs affected by both Aβ and tau, it may help researchers to identify novel molecular mechanisms in the Aβ-tau axis.

To verify whether ADLP^APT^ mice and its proteome data could be applied to further AD researches, we performed a comparative analysis with existing AD proteome datasets. First, the proteome data of ADLP^APT^ mice were compared with that of human AD brain generated by Seyfried and Levey [50]. As a result, approximately 92% of the identified proteins were overlapped with the human AD proteome and approximately 30% of significantly regulated proteins in human AD data showed identical expression patterns with those of ADLP^APT^ mice (*p*-value <0.05 in both studies, respectively). We also assessed the commonality between our mouse model and the previously established 3xTg AD mouse model [73–75]. Notably, the comparative analysis indicated that our DEPs were largely different from those of other studies of 3xTg AD mice (data not shown). Most of our DEPs were neither detected nor showed consistent expression patterns in other studies. This may be attributed to the fact that the results of other studies were generated from 2-DIGE analysis [73] or the vesicular proteome isolated from the forebrain [74]. To the best of our knowledge, this study is the first to perform proteomic analysis in hippocampi developing both amyloid and NFT pathologies. These results indicate that the pathological relevance of our mouse model for AD research is valid at the protein level and in severe hippocampal pathologies.

## Conclusion

We herein described a novel mouse model for AD, namely ADLP^APT^ mouse. This model exhibited a rapid progression of AD pathology, including neuronal death and an accelerated NFT pathology that are particularly accelerated by the amyloid pathology. Thus, this model recapitulated the progression of human AD. Using LC-MS with 10-plex TMT isobaric labeling, we further constructed a hippocampal proteome database of ADLP mouse models. We used this database to understand the biological functions that were altered in each transgenic mouse model. This proteomic database also offers extensive resource of various molecular phenotypes in response to amyloid and NFT pathologies. Based on the bioinformatics analysis, we propose potential molecular targets for mechanistic studies and therapeutic development based on the proteomic database generated from our novel animal model.

## Additional files

Additional file 1: Supplementary Methods. (DOCX 31 kb)

Additional file 2: **Figure S1.** Pathological characterization of a novel animal model of Alzheimer’s disease. **Figure S2.** Activated neuroinflammation in a novel animal model of Alzheimer’s disease. **Figure S3.** TMT-based protein quantification strategy. **Figure S4.** The comparative analysis between ADLP^APT^ and other AD proteome datasets. **Figure S5.** The quality assessment of MS analysis. **Figure S6.** The expression levels of exclusive DEPs^APT^ in other ADLP mice. **Figure S7.** Longitudinal expression changes of kinases involved in phosphorylation of tau protein. (PPTX 2370 kb)

Additional file 3: Table S1. All identified protein groups. (XLSX 1130 kb)

Additional file 4: Table S2. All quantified protein groups. (XLSX 4540 kb)

Additional file 5: Table S3. Significantly regulated proteins from t-test. (XLSX 170 kb)

Additional file 6: Table S4. DEPs from ANOVA test. (XLSX 391 kb)

Additional file 7: Table S5. Canonical pathways enriched by IPA analysis. (XLSX 18 kb)

Additional file 8: Table S6. DEPs in ADLP^APT^ mice, DEPs^APT^. (XLSX 195 kb)

Additional file 9: Table S7. GO analysis results in ADLP^APT^ mice. (XLSX 41 kb)

Additional file 10: Table S8. IPA analysis results in ADLP^APT^ mice. (XLSX 53 kb)

Additional file 11: Table S9. Exclusive DEPs^APT^. (XLSX 31 kb)

Additional file 12: Table S10. GO analysis of commonly significant proteins in human and ADLP^APT^. (XLSX 13 kb)

## Abbreviations

ADLP: Alzheimer’s disease-like pathology
APP: Amyloid precursor protein
Aβ: Amyloid beta
BPRP: Basic pH reverse phase
DEPs: Differentially expressed proteins
GO: Gene ontology
IPA: Ingenuity pathway analysis
LC-MS: Liquid chromatography - Mass spectrometry
LTP: Long term potentiation
NFT: Neurofibrillary tangle
PPI: Protein-protein interaction
TMT: Tandem mass tag
