# Supplementary material for: Molecular and functional signatures in a novel Alzheimer’s disease mouse model assessed by quantitative proteomics
Source: Mol Neurodegener. 2018 Jan 16;13:2. doi: 10.1186/s13024-017-0234-4 (PMC5771139; doi:10.1186/s13024-017-0234-4)
Supplement: Supplementary file 1 — Supplementary Methods. (DOCX 31 kb) [file 13024_2017_234_MOESM1_ESM.docx]

**Additional File 1 : Supplementary methods**

**Tissue Preparation for Proteomic Analysis**

Mouse hippocampus tissues were resected and subjected to the previously described sample preparation method with some modifications (1, 2). First of all, both sides of hippocampus tissue samples were washed three times with sterilized phosphate buffered saline (PBS). Washed tissues were lysed with 300 μl of lysis buffer (4% SDS, 1 mM TCEP in 0.1 M TEAB pH 8.5), followed by sonication. Protein concentration was measured using a BCA reducing agent compatibility assay kit (Thermo Scientific, Rockford, IL, USA). A pooled protein sample was prepared by taking 50 μg aliquots from all sample.

Protein digestion was performed by a combined approach including acetone precipitation and FASP procedure described previously (1, 2). Prior to the digestion step, 300 μg of protein was precipitated overnight by adding a five-fold volume of ice-cold acetone. Precipitated proteins were dissolved in 30 μl denaturation buffer (4% SDS and 100 mM DTT in 0.1M TEAB pH 8.5). After heating at 99℃ for 15 min, denatured proteins were loaded onto 30 kDa spin filter (Merck Millipore, Darmstadt, Germany). The buffer was exchanged three times with UA solution (8 M UREA in 0.1 M TEAB pH 8.5) by centrifugation at 14,000 *g*. After removal of SDS, cysteine alkylation was accomplished through the addition of alkylation buffer (50 mM IAA, 8 M UREA in 0.1 M Tris-HCl pH 8.5) for 1 hour at room temperature in the dark. UA buffer was exchanged with TEAB buffer (40 mM TEAB pH 8.5). The proteins were digested with trypsin (enzyme-to-substrate ratio [w/w] of 1:100) and 5% ACN at 37℃ overnight. The digested peptides were collected by centrifugation and the peptide concentrations were measured by tryptophan fluorescence emission at 350 nm using an excitation wavelength of 295 nm (3). The pooled protein sample and the external standard, ovalbumin, sample were digested in the same manner.

**Tandem Mass Tags (TMT) Labeling**

Due to the limited number of channel, we decided to distribute the 36 samples (4 mouse models * 3 age-points * biological triplicate) to four TMT experimental sets. After all the samples were randomly disposed using Excel, each experiment set had 9 individual samples and a common pooled sample (Additional file 2: Figure S3A). Prior to the labeling step, each 50 μg peptide sample was spiked with a uniform volume of ovalbumin. Then, 40 mM TEAB buffer was added to each sample to equalize the volume. To remove the error derived by the reagent, we dissolved one set of TMT reagent and spiked equally to each experimental set. TMT reagents (0.8 mg) were dissolved in 110 μl of anhydrous ACN, of which 25 μl was added to identical channels in 4 experimental sets. Then, 35 μl of ACN was added to achieve final concentration of 30%. Following incubation at room temperature for 1 hour, the reaction was quenched with hydroxylamine to a final concentration of 0.3% (v/v). The TMT-labeled samples were pooled at a ratio of 1:1 across all samples. The sample was lyophilized to near dryness and subjected to the desalting procedure.

**Sample Desalting and Basic pH Revered Phase (BPRP) Peptide Fractionation**

The TMT-labeled peptide samples were desalted using HLB OASIS column according to the manufacturer’s instruction. Consequently, Basic pH Reversed-Phase peptide fractionation was performed using Agilent 1260 bioinert HPLC (Agilent, Santa Clara, CA) equipped with an Agilent 300 Extended-C18 column (4.6 mm I.D x 15 cm long, 5 μm C18 particle). Peptide samples were separated with a 60 min linear gradient from 5% to 40% ACN in 15 mM ammonium hydroxide at a flow rate of 1 mL/min. The sample was fractionated into a total of 96 fractions, which were non-contiguously concatenated into 12 fractions. The fractions were lyophilized and stored at -80℃ until MS analysis.

**RP-nano LC-ESI-MS/MS Analysis**

The fractionated peptide samples were analyzed by LC-MS system which was a combination of an Easy-nLC 1000 (Thermo Fisher Scientific, Waltham, MA) coupled to a nano-electrospray ion source (Thermo Fisher Scientific, Waltham, MA) on a Q-Exactive mass spectrometer (Thermo Fisher Scientific, Waltham, MA), according to our established protocol (1, 2). Peptides were separated on the 2-column setup with a trap column (Thermo Fisher Scientific, 75 μm I.D. x 2 cm long, 3 μm Acclaim PepMap100 C18 beads) and an analytic column (75 μm I.D. x 50 cm long, 3 μm ReproSil-Pur-AQ C18 beads). Prior to sample injection, dried peptide samples were re-dissolved in solvent A (2% ACN and 0.1% Formic acid). The peptide samples were separated with a 240-minutes nonlinear gradient from 8% to 60% solvent B (100% ACN and 0.1% Formic acid) in all samples. The spray voltage was 2.2 kV in positive ion mode, and the temperature of the heated capillary was set to 320 °C. Mass spectra were acquired in a data-dependent manner using a top 15 method on a Q-Exactive. Xcaliber software version 2.5 was used to collect MS data. The Orbitrap analyzer scanned precursor ions with a mass range of 300–1,650 m/z with 70,000 resolution at m/z 200. The automatic gain control (AGC) target value was 3 x 10^6^ and the isolation window for MS/MS was 1.2 m/z. HCD scans were acquired at a resolution of 35,000 and 32 normalized collision energy (NCE). The AGC target value for MS/MS was 2 x 10^5^. The maximum ion injection time for the survey scan and MS/MS scan was 30 ms and 120 ms, respectively. Dynamic exclusion was enabled with an exclusion period of 40 s.

**Computational MS Data Analysis**

Proteome Discoverer version 2.1 (Thermo Fisher Scientific, Waltham, MA) was used to perform a database search. The tandem mass spectra search was performed by the SEQUEST HT algorithm against the Uniprot *Mus musculus* database (December, 2015 released; 82,074 protein entries, http://www.uniprot.org), which included additional proteins: mutated human *APP*, *PSEN1*, *MAPT*, and chicken ovalbumin. The database search was conducted according to the target-decoy search strategy. The search parameters were: full enzyme digest using trypsin (After KR/−) up to 2 missed cleavages; a precursor ion mass tolerance of 15 ppm (monoisotopic mass); a fragment ion mass tolerance of 0.02 Da (monoisotopic mass); static modifications of 229.163 Da on lysine residues and peptide N-termini for TMT and 57.02 Da on cysteine residues for carbamidomethylation; and dynamic modifications of 42.01 Da for protein N-term acetylation and 15.99 Da for methionine oxidation. Confidence criteria were set to a false discovery rate (FDR) of less than 1% at both the peptide and protein level. Proteins were quantified by calculating reporter ion intensities using the “Reporter Ions Quantifier” node in Proteome Discoverer. The co-isolation threshold was set to 50%. The MS-based proteomics data of all identified peptides and proteins list have been deposited in the ProteomeXchange Consortium (http://proteomecentral.proteomexchange.org) via the PRIDE partner repository (4): dataset identifier PXD006214.

1. Han, D., S. Moon, Y. Kim, J. Kim, J. Jin, and Y. Kim, In-depth proteomic analysis of mouse microglia using a combination of FASP and StageTip-based, high pH, reversed-phase fractionation. *Proteomics*, (2013). 13(20): p. 2984-8.

2. Han, D., J. Jin, J. Woo, H. Min, and Y. Kim, Proteomic analysis of mouse astrocytes and their secretome by a combination of FASP and StageTip-based, high pH, reversed-phase fractionation. *Proteomics*, (2014). 14(13-14): p. 1604-9.

3. Kulak, N.A., G. Pichler, I. Paron, N. Nagaraj, and M. Mann, Minimal, encapsulated proteomic-sample processing applied to copy-number estimation in eukaryotic cells. *Nat Methods*, (2014). 11(3): p. 319-24.

4. Vizcaino, J.A., E.W. Deutsch, R. Wang, A. Csordas, F. Reisinger, D. Rios, J.A. Dianes, Z. Sun, T. Farrah, N. Bandeira, P.A. Binz, I. Xenarios, M. Eisenacher, G. Mayer, L. Gatto, A. Campos, R.J. Chalkley, H.J. Kraus, J.P. Albar, S. Martinez-Bartolome, R. Apweiler, G.S. Omenn, L. Martens, A.R. Jones, and H. Hermjakob, ProteomeXchange provides globally coordinated proteomics data submission and dissemination. *Nat Biotechnol*, (2014). 32(3): p. 223-6.
