# Supplementary material for: Molecular and functional signatures in a novel Alzheimer’s disease mouse model assessed by quantitative proteomics
Source: Mol Neurodegener. 2018 Jan 16;13:2. doi: 10.1186/s13024-017-0234-4 (PMC5771139; doi:10.1186/s13024-017-0234-4)
Supplement: Supplementary file 2 — Figure S1. Pathological characterization of a novel animal model of Alzheimer’s disease. Figure S2. Activated neuroinflammation in a novel animal model of Alzheimer’s disease. Figure S3. TMT-based protein quantification strategy. Figure S4. The comparative analysis between ADLPAPT and other AD proteome datasets. Figure S5. The quality assessment of MS analysis. Figure S6. The expression levels of exclusive DEPsAPT in other ADLP mice. Figure S7. Longitudinal expression changes of kinases involved in phosphorylation of tau protein. (PPTX 2370 kb) [file 13024_2017_234_MOESM2_ESM.pptx]

## Slide 1
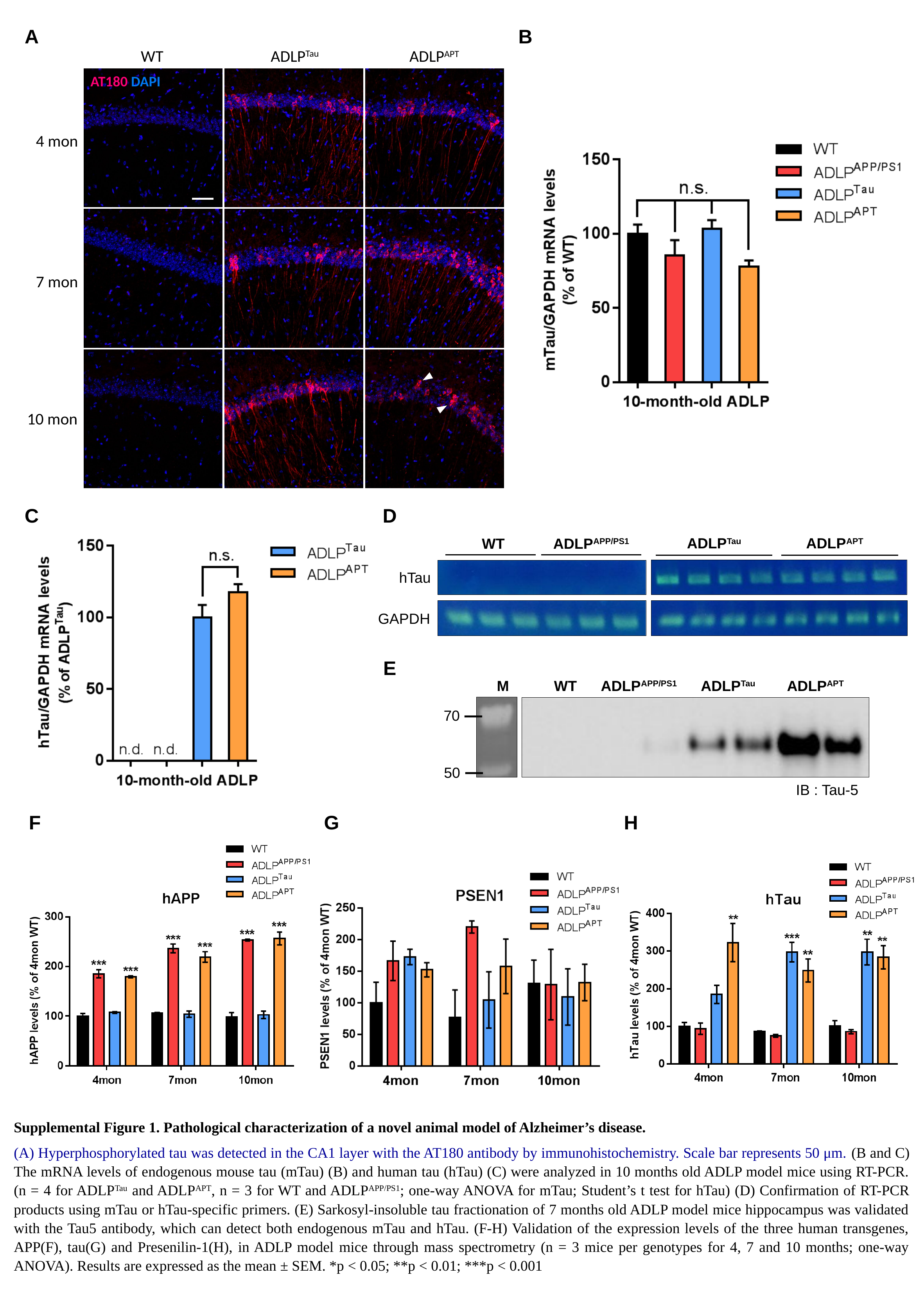

B
A
WT
ADLPTau
ADLPAPT
4 mon
7 mon
10 mon
AT180 DAPI
C
D
 ADLPTau ADLPAPT
 WT ADLPAPP/PS1
hTau
GAPDH
E
M WT ADLPAPP/PS1 ADLPTau ADLPAPT
_
70
_
50
IB : Tau-5
F
G
H
Supplemental Figure 1. Pathological characterization of a novel animal model of Alzheimer’s disease.
(A) Hyperphosphorylated tau was detected in the CA1 layer with the AT180 antibody by immunohistochemistry. Scale bar represents 50 μm. (B and C) The mRNA levels of endogenous mouse tau (mTau) (B) and human tau (hTau) (C) were analyzed in 10 months old ADLP model mice using RT-PCR. (n = 4 for ADLPTau and ADLPAPT, n = 3 for WT and ADLPAPP/PS1; one-way ANOVA for mTau; Student’s t test for hTau) (D) Confirmation of RT-PCR products using mTau or hTau-specific primers. (E) Sarkosyl-insoluble tau fractionation of 7 months old ADLP model mice hippocampus was validated with the Tau5 antibody, which can detect both endogenous mTau and hTau. (F-H) Validation of the expression levels of the three human transgenes, APP(F), tau(G) and Presenilin-1(H), in ADLP model mice through mass spectrometry (n = 3 mice per genotypes for 4, 7 and 10 months; one-way ANOVA). Results are expressed as the mean ± SEM. *p < 0.05; **p < 0.01; ***p < 0.001

## Slide 2
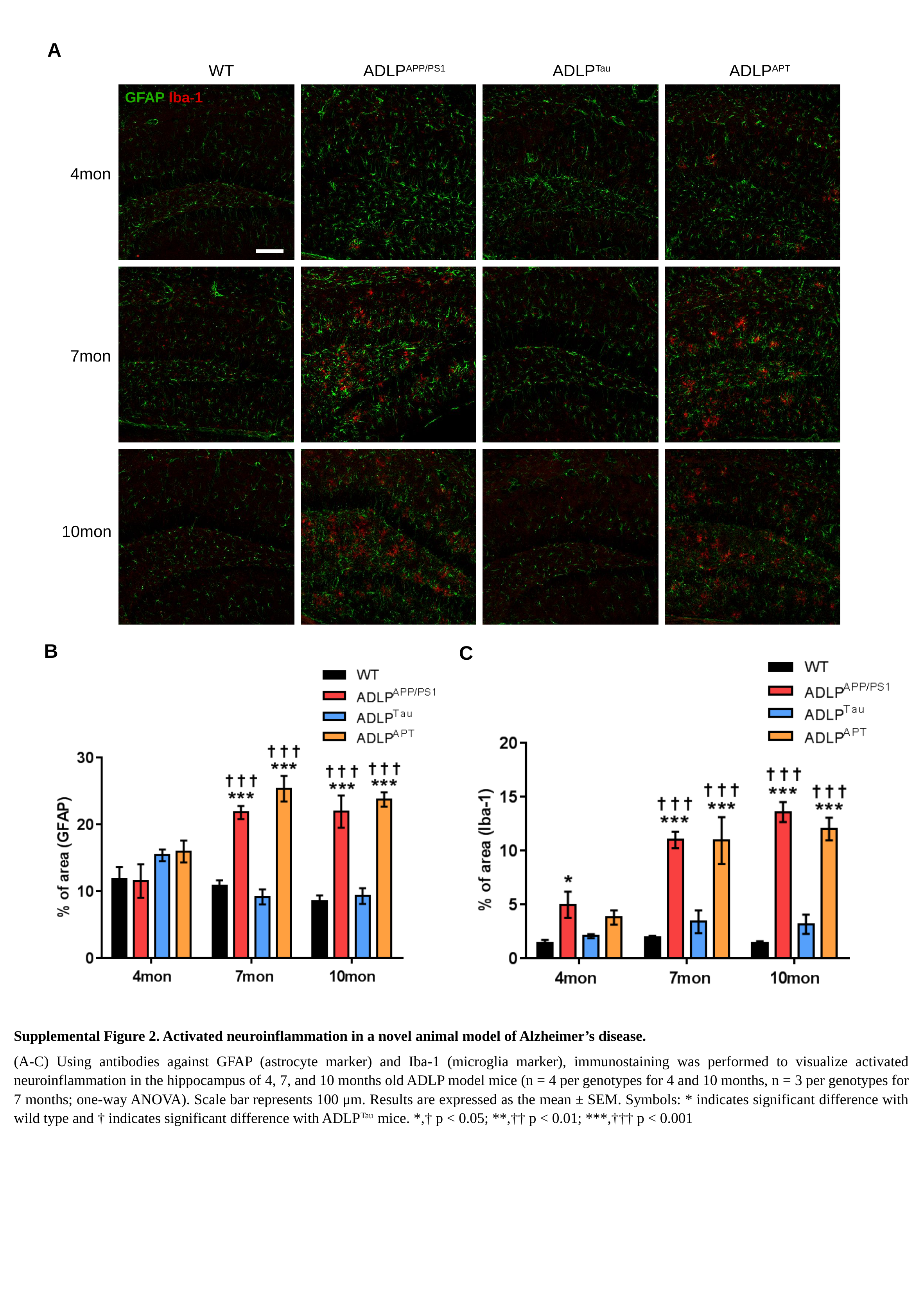

A
WT ADLPAPP/PS1 ADLPTau ADLPAPT
GFAP Iba-1
4mon
7mon
10mon
B
C
Supplemental Figure 2. Activated neuroinflammation in a novel animal model of Alzheimer’s disease.
(A-C) Using antibodies against GFAP (astrocyte marker) and Iba-1 (microglia marker), immunostaining was performed to visualize activated neuroinflammation in the hippocampus of 4, 7, and 10 months old ADLP model mice (n = 4 per genotypes for 4 and 10 months, n = 3 per genotypes for 7 months; one-way ANOVA). Scale bar represents 100 μm. Results are expressed as the mean ± SEM. Symbols: * indicates significant difference with wild type and † indicates significant difference with ADLPTau mice. *,† p < 0.05; **,†† p < 0.01; ***,††† p < 0.001

## Slide 3
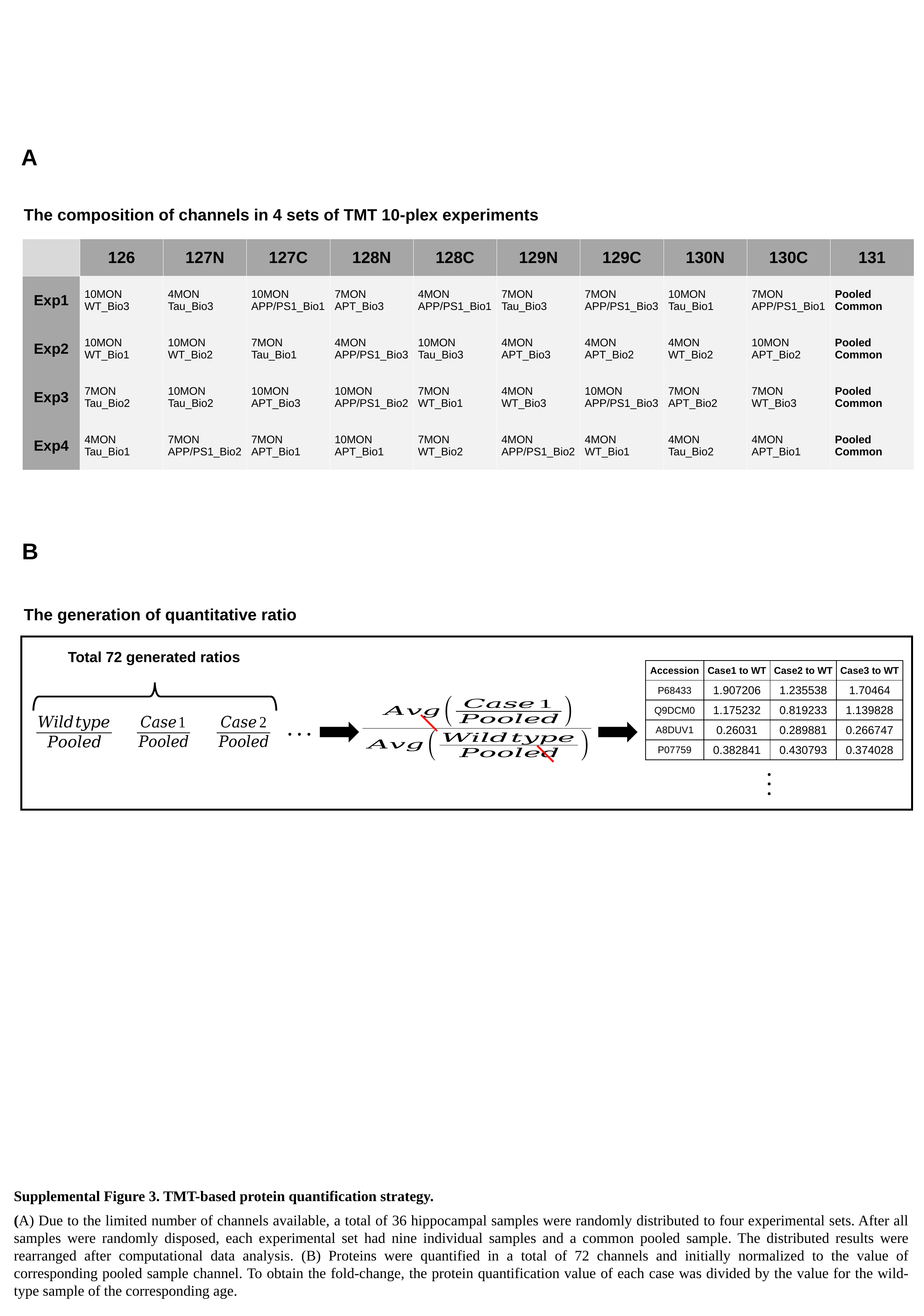

A
The composition of channels in 4 sets of TMT 10-plex experiments
| | 126 | 127N | 127C | 128N | 128C | 129N | 129C | 130N | 130C | 131 |
| --- | --- | --- | --- | --- | --- | --- | --- | --- | --- | --- |
| Exp1 | 10MON WT\_Bio3 | 4MON Tau\_Bio3 | 10MON APP/PS1\_Bio1 | 7MON APT\_Bio3 | 4MON APP/PS1\_Bio1 | 7MON Tau\_Bio3 | 7MON APP/PS1\_Bio3 | 10MON Tau\_Bio1 | 7MON APP/PS1\_Bio1 | Pooled Common |
| Exp2 | 10MON WT\_Bio1 | 10MON WT\_Bio2 | 7MON Tau\_Bio1 | 4MON APP/PS1\_Bio3 | 10MON Tau\_Bio3 | 4MON APT\_Bio3 | 4MON APT\_Bio2 | 4MON WT\_Bio2 | 10MON APT\_Bio2 | Pooled Common |
| Exp3 | 7MON Tau\_Bio2 | 10MON Tau\_Bio2 | 10MON APT\_Bio3 | 10MON APP/PS1\_Bio2 | 7MON WT\_Bio1 | 4MON WT\_Bio3 | 10MON APP/PS1\_Bio3 | 7MON APT\_Bio2 | 7MON WT\_Bio3 | Pooled Common |
| Exp4 | 4MON Tau\_Bio1 | 7MON APP/PS1\_Bio2 | 7MON APT\_Bio1 | 10MON APT\_Bio1 | 7MON WT\_Bio2 | 4MON APP/PS1\_Bio2 | 4MON WT\_Bio1 | 4MON Tau\_Bio2 | 4MON APT\_Bio1 | Pooled Common |
B
The generation of quantitative ratio
Total 72 generated ratios
| Accession | Case1 to WT | Case2 to WT | Case3 to WT |
| --- | --- | --- | --- |
| P68433 | 1.907206 | 1.235538 | 1.70464 |
| Q9DCM0 | 1.175232 | 0.819233 | 1.139828 |
| A8DUV1 | 0.26031 | 0.289881 | 0.266747 |
| P07759 | 0.382841 | 0.430793 | 0.374028 |
. . .
. . .
Supplemental Figure 3. TMT-based protein quantification strategy.
(A) Due to the limited number of channels available, a total of 36 hippocampal samples were randomly distributed to four experimental sets. After all samples were randomly disposed, each experimental set had nine individual samples and a common pooled sample. The distributed results were rearranged after computational data analysis. (B) Proteins were quantified in a total of 72 channels and initially normalized to the value of corresponding pooled sample channel. To obtain the fold-change, the protein quantification value of each case was divided by the value for the wild-type sample of the corresponding age.

## Slide 4
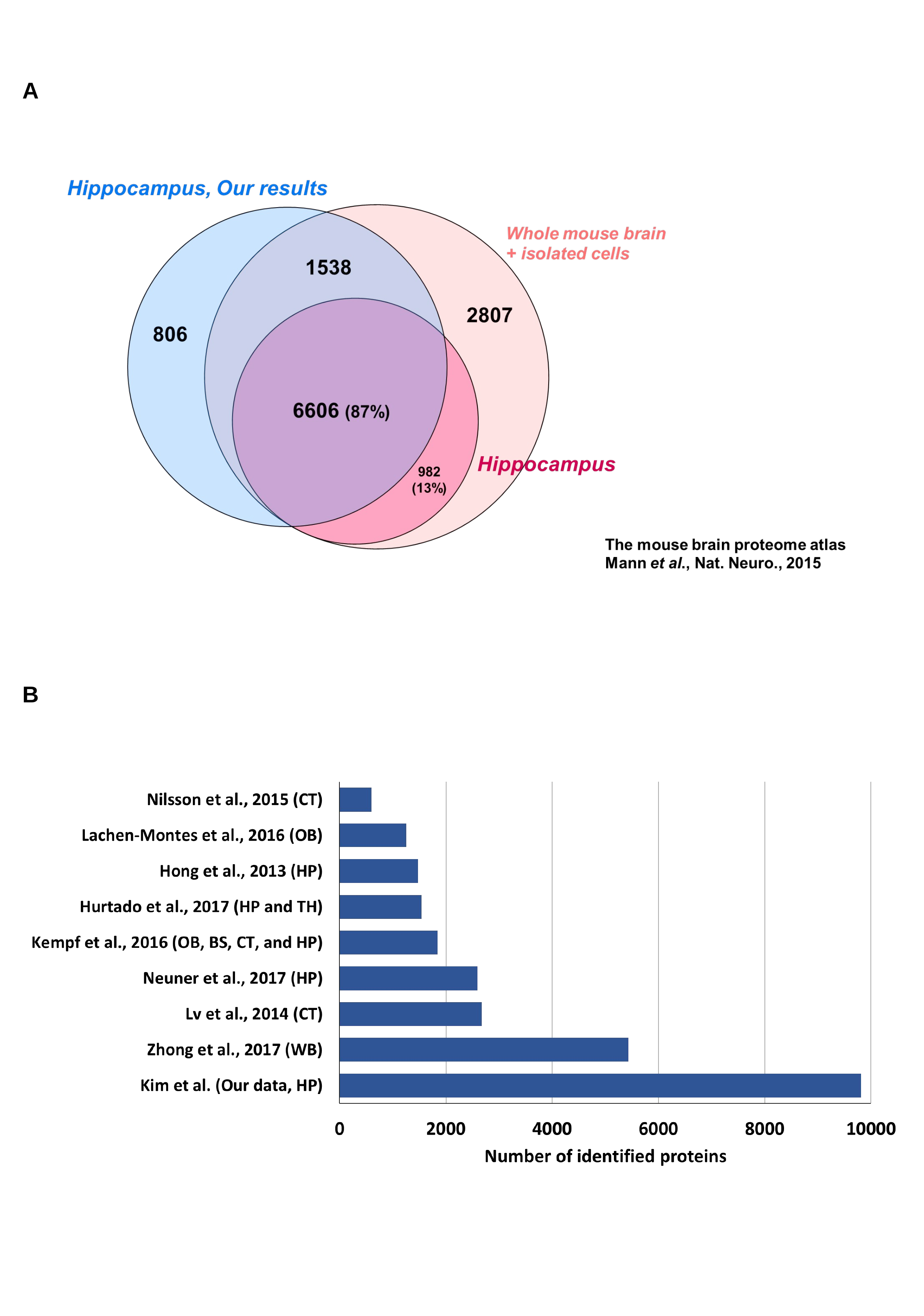

A
B

## Slide 5
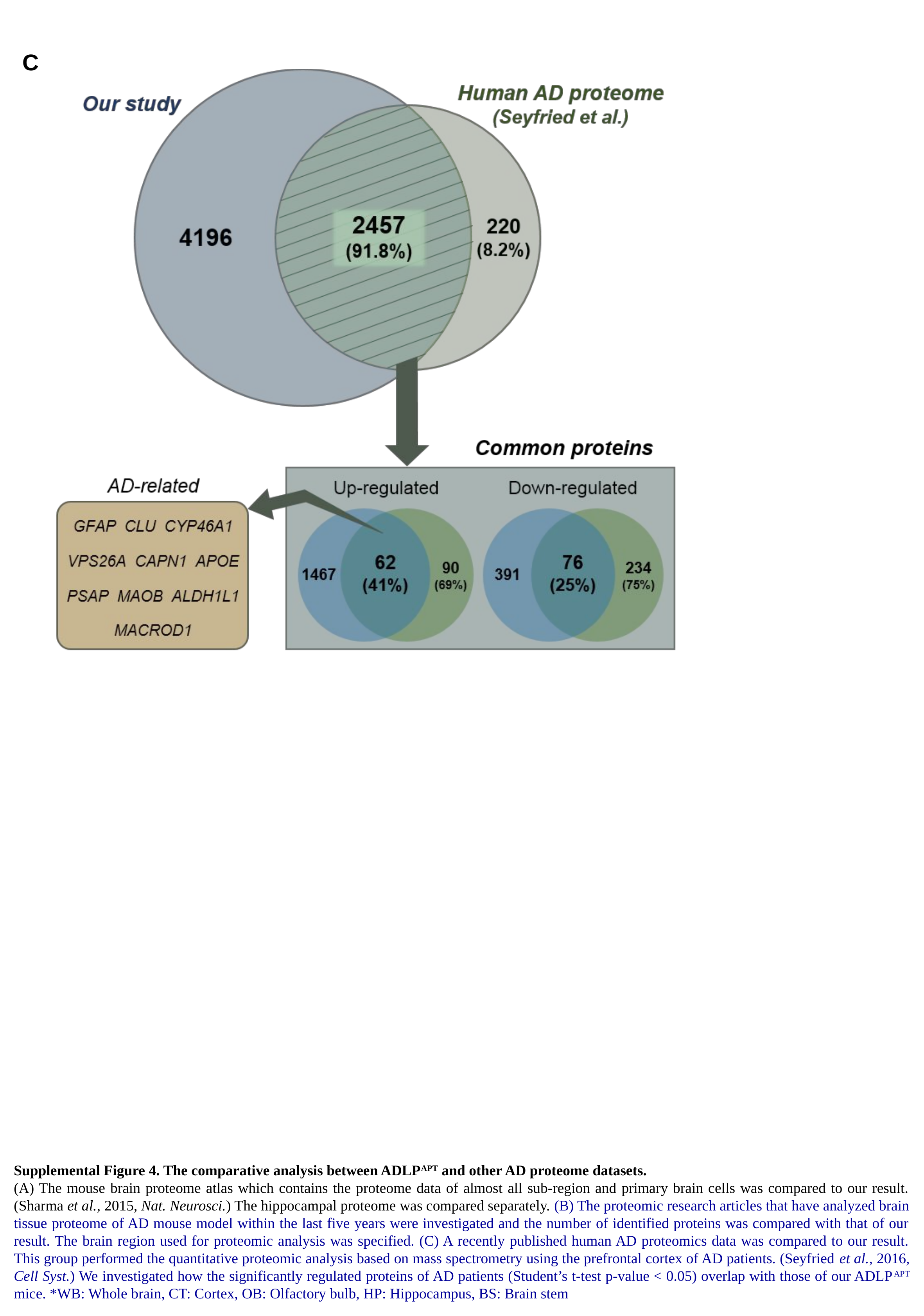

C
Supplemental Figure 4. The comparative analysis between ADLPAPT and other AD proteome datasets.
(A) The mouse brain proteome atlas which contains the proteome data of almost all sub-region and primary brain cells was compared to our result. (Sharma et al., 2015, Nat. Neurosci.) The hippocampal proteome was compared separately. (B) The proteomic research articles that have analyzed brain tissue proteome of AD mouse model within the last five years were investigated and the number of identified proteins was compared with that of our result. The brain region used for proteomic analysis was specified. (C) A recently published human AD proteomics data was compared to our result. This group performed the quantitative proteomic analysis based on mass spectrometry using the prefrontal cortex of AD patients. (Seyfried et al., 2016, Cell Syst.) We investigated how the significantly regulated proteins of AD patients (Student’s t-test p-value < 0.05) overlap with those of our ADLPAPT mice. *WB: Whole brain, CT: Cortex, OB: Olfactory bulb, HP: Hippocampus, BS: Brain stem

## Slide 6
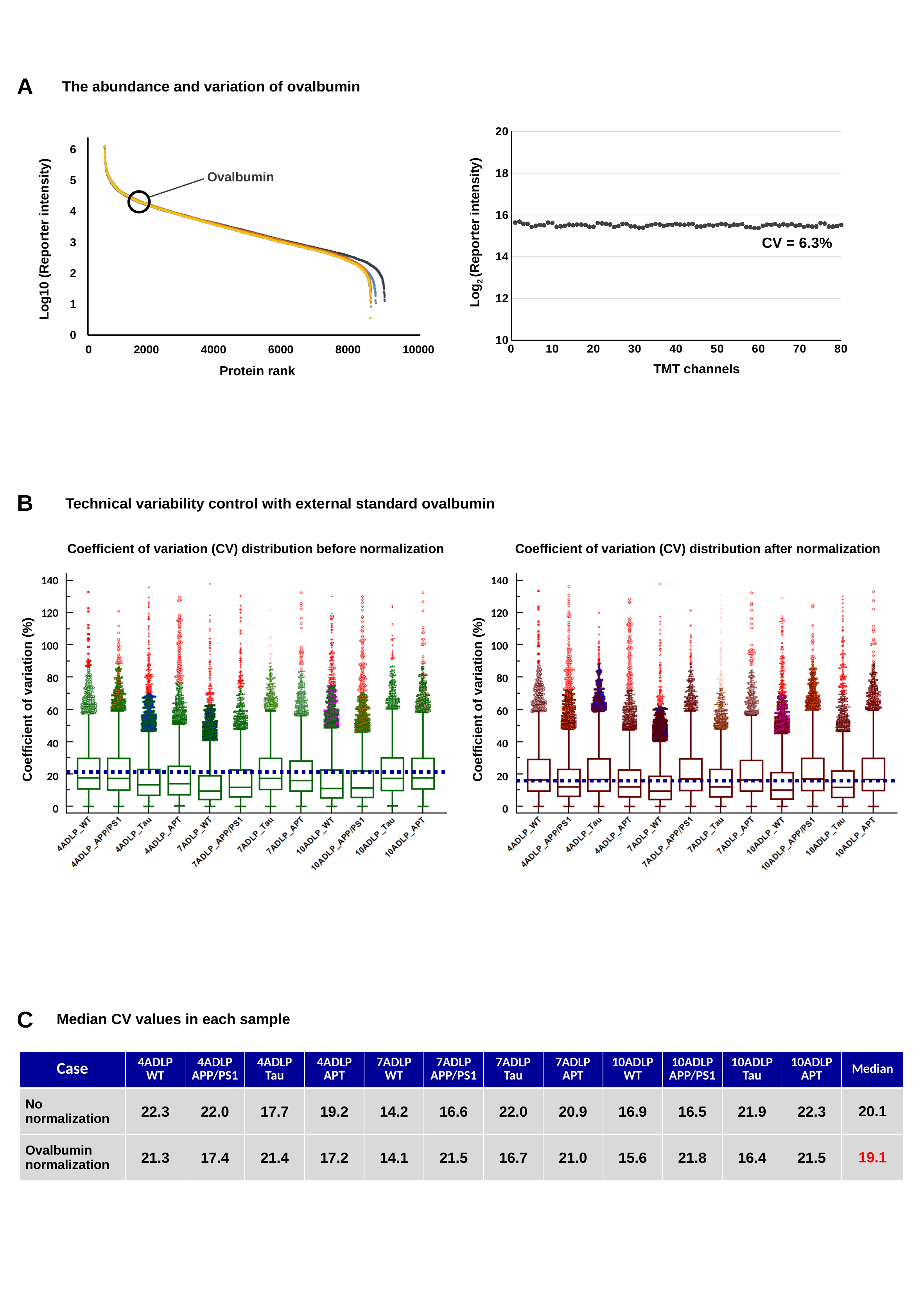

A
The abundance and variation of ovalbumin
### Chart
| Category | |
|---|---|
6
5
4
3
2
1
0
Ovalbumin
Log2 (Reporter intensity)
Log10 (Reporter intensity)
CV = 6.3%
 0 2000 4000 6000 8000 10000
TMT channels
Protein rank
B
Technical variability control with external standard ovalbumin
Coefficient of variation (CV) distribution before normalization
Coefficient of variation (CV) distribution after normalization
140
120
100
80
60
40
20
0
140
120
100
80
60
40
20
0
Coefficient of variation (%)
Coefficient of variation (%)
C
Median CV values in each sample
| Case | 4ADLP WT | 4ADLP APP/PS1 | 4ADLP Tau | 4ADLP APT | 7ADLP WT | 7ADLP APP/PS1 | 7ADLP Tau | 7ADLP APT | 10ADLP WT | 10ADLP APP/PS1 | 10ADLP Tau | 10ADLP APT | Median |
| --- | --- | --- | --- | --- | --- | --- | --- | --- | --- | --- | --- | --- | --- |
| No normalization | 22.3 | 22.0 | 17.7 | 19.2 | 14.2 | 16.6 | 22.0 | 20.9 | 16.9 | 16.5 | 21.9 | 22.3 | 20.1 |
| Ovalbumin normalization | 21.3 | 17.4 | 21.4 | 17.2 | 14.1 | 21.5 | 16.7 | 21.0 | 15.6 | 21.8 | 16.4 | 21.5 | 19.1 |

## Slide 7
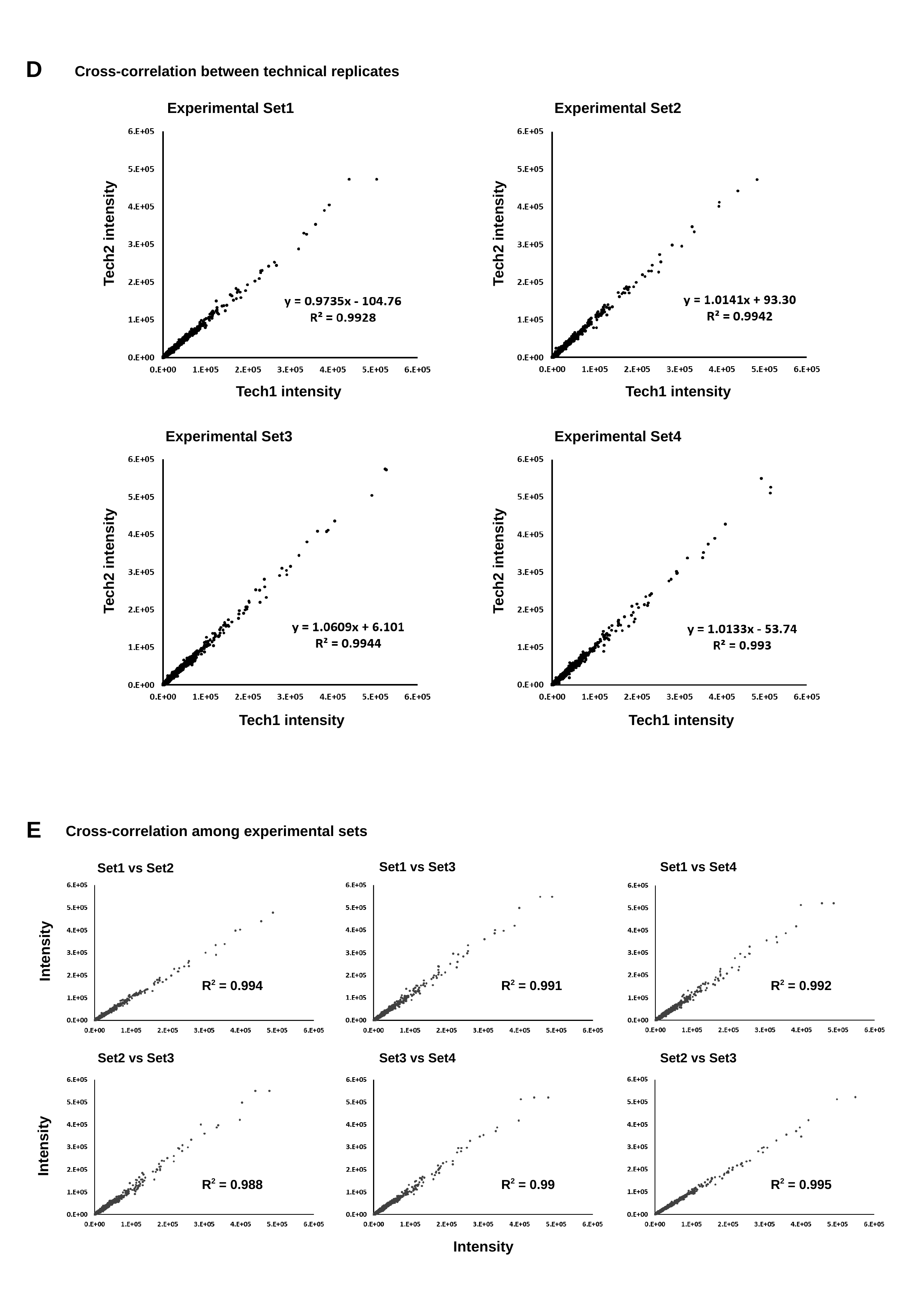

D
Cross-correlation between technical replicates
Experimental Set1
Experimental Set2
Tech2 intensity
Tech2 intensity
Tech1 intensity
Tech1 intensity
Experimental Set3
Experimental Set4
Tech2 intensity
Tech2 intensity
Tech1 intensity
Tech1 intensity
E
Cross-correlation among experimental sets
Set1 vs Set4
Set1 vs Set3
Set1 vs Set2
Intensity
R2 = 0.994
R2 = 0.991
R2 = 0.992
Set2 vs Set3
Set3 vs Set4
Set2 vs Set3
Intensity
R2 = 0.988
R2 = 0.99
R2 = 0.995
Intensity

## Slide 8
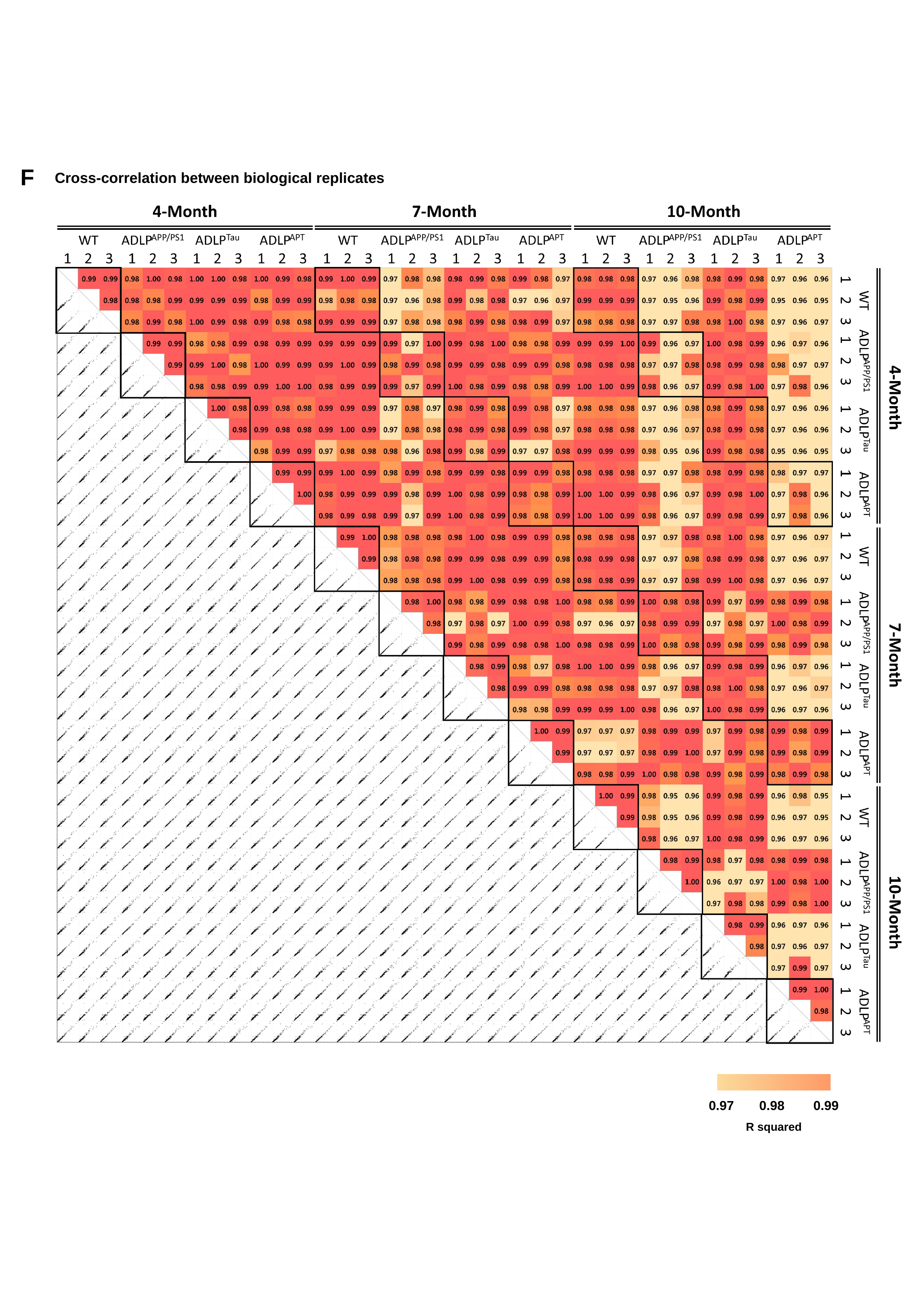

F
Cross-correlation between biological replicates
0.97 0.98 0.99
R squared

## Slide 9
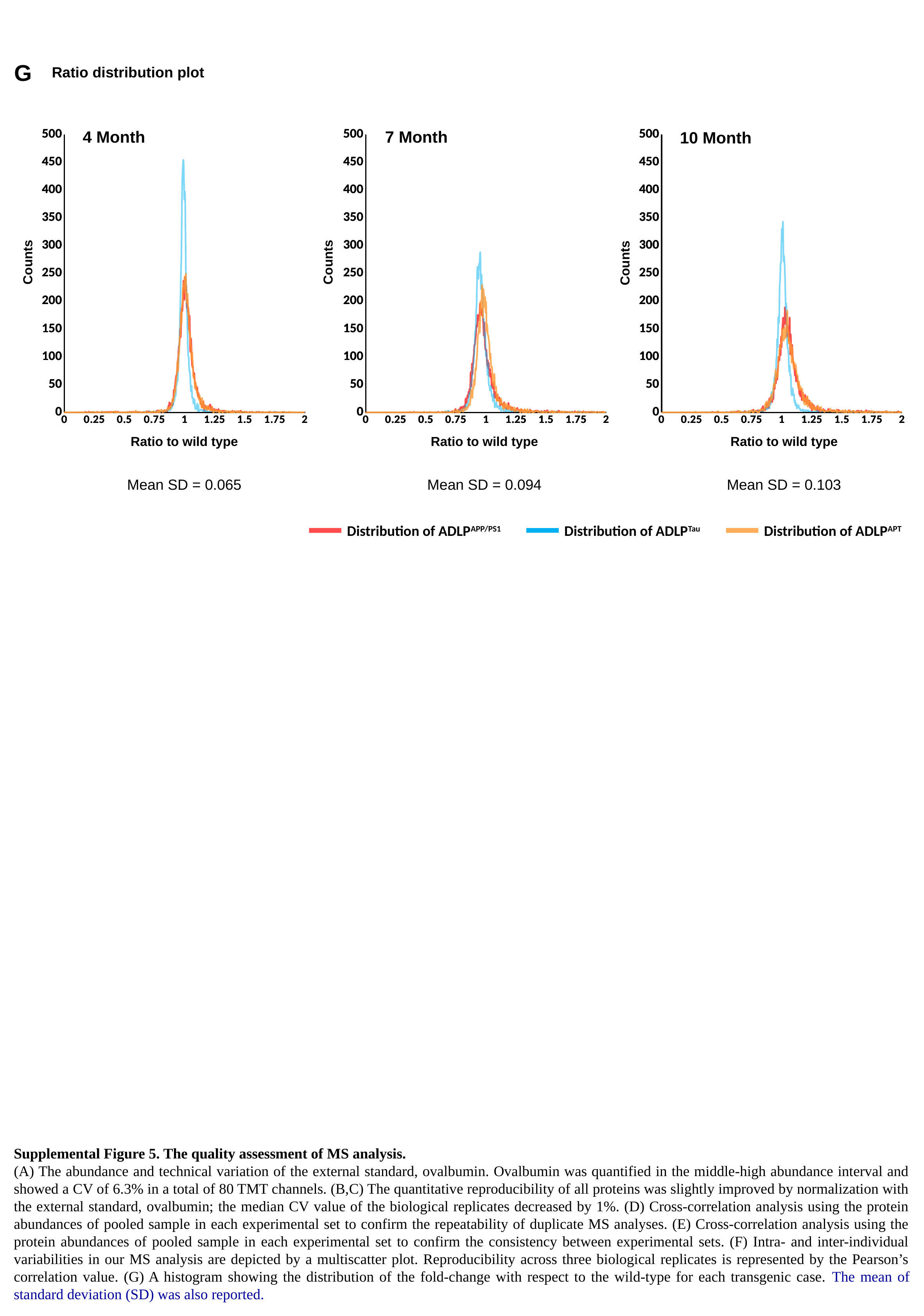

G
Ratio distribution plot
### Chart
| Category | 4Tau_FC_0 |
|---|---|
### Chart
| Category | 4APT_FC_0 |
|---|---|
### Chart
| Category | 4Ab_FC_0 |
|---|---|
### Chart
| Category | 7APT_FC_0 |
|---|---|
### Chart
| Category | 7Tau_FC_0 |
|---|---|
### Chart
| Category | 7Ab_FC_0 |
|---|---|
### Chart
| Category | 10Tau_FC_0 |
|---|---|
### Chart
| Category | 10APT_FC_0 |
|---|---|
### Chart
| Category | 10Ab_FC_0 |
|---|---|4 Month
7 Month
10 Month
Counts
Counts
Counts
Ratio to wild type
Ratio to wild type
Ratio to wild type
Mean SD = 0.094
Mean SD = 0.103
Mean SD = 0.065
Distribution of ADLPAPP/PS1
Distribution of ADLPTau
Distribution of ADLPAPT
Supplemental Figure 5. The quality assessment of MS analysis.
(A) The abundance and technical variation of the external standard, ovalbumin. Ovalbumin was quantified in the middle-high abundance interval and showed a CV of 6.3% in a total of 80 TMT channels. (B,C) The quantitative reproducibility of all proteins was slightly improved by normalization with the external standard, ovalbumin; the median CV value of the biological replicates decreased by 1%. (D) Cross-correlation analysis using the protein abundances of pooled sample in each experimental set to confirm the repeatability of duplicate MS analyses. (E) Cross-correlation analysis using the protein abundances of pooled sample in each experimental set to confirm the consistency between experimental sets. (F) Intra- and inter-individual variabilities in our MS analysis are depicted by a multiscatter plot. Reproducibility across three biological replicates is represented by the Pearson’s correlation value. (G) A histogram showing the distribution of the fold-change with respect to the wild-type for each transgenic case. The mean of standard deviation (SD) was also reported.

## Slide 10
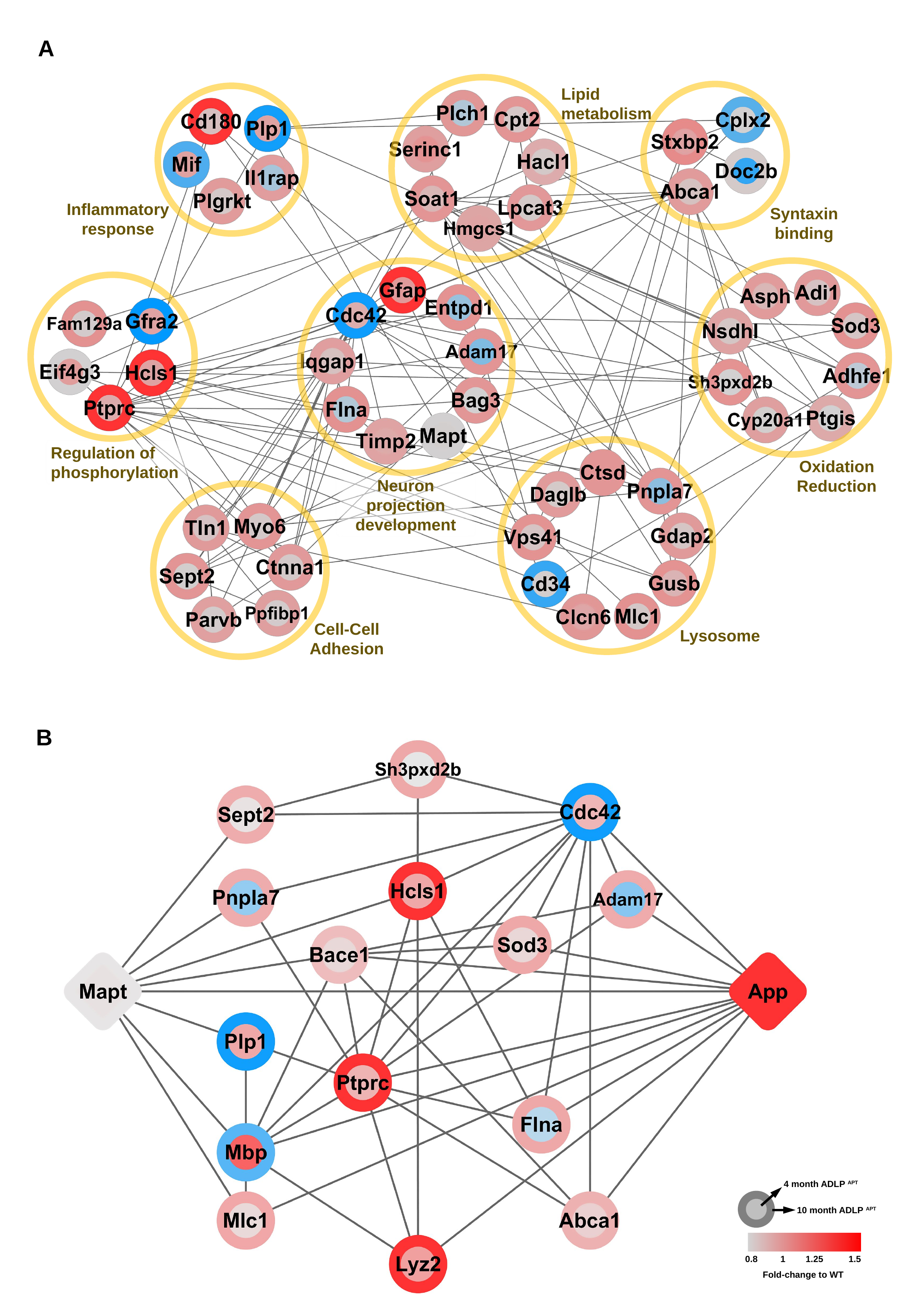

A
Lipid metabolism
Inflammatory response
Syntaxin binding
Regulation of phosphorylation
Oxidation
Reduction
Neuron projection development
Cell-Cell
Adhesion
Lysosome
B
4 month ADLP APT
10 month ADLP APT
0.8 1 1.25 1.5
Fold-change to WT

## Slide 11
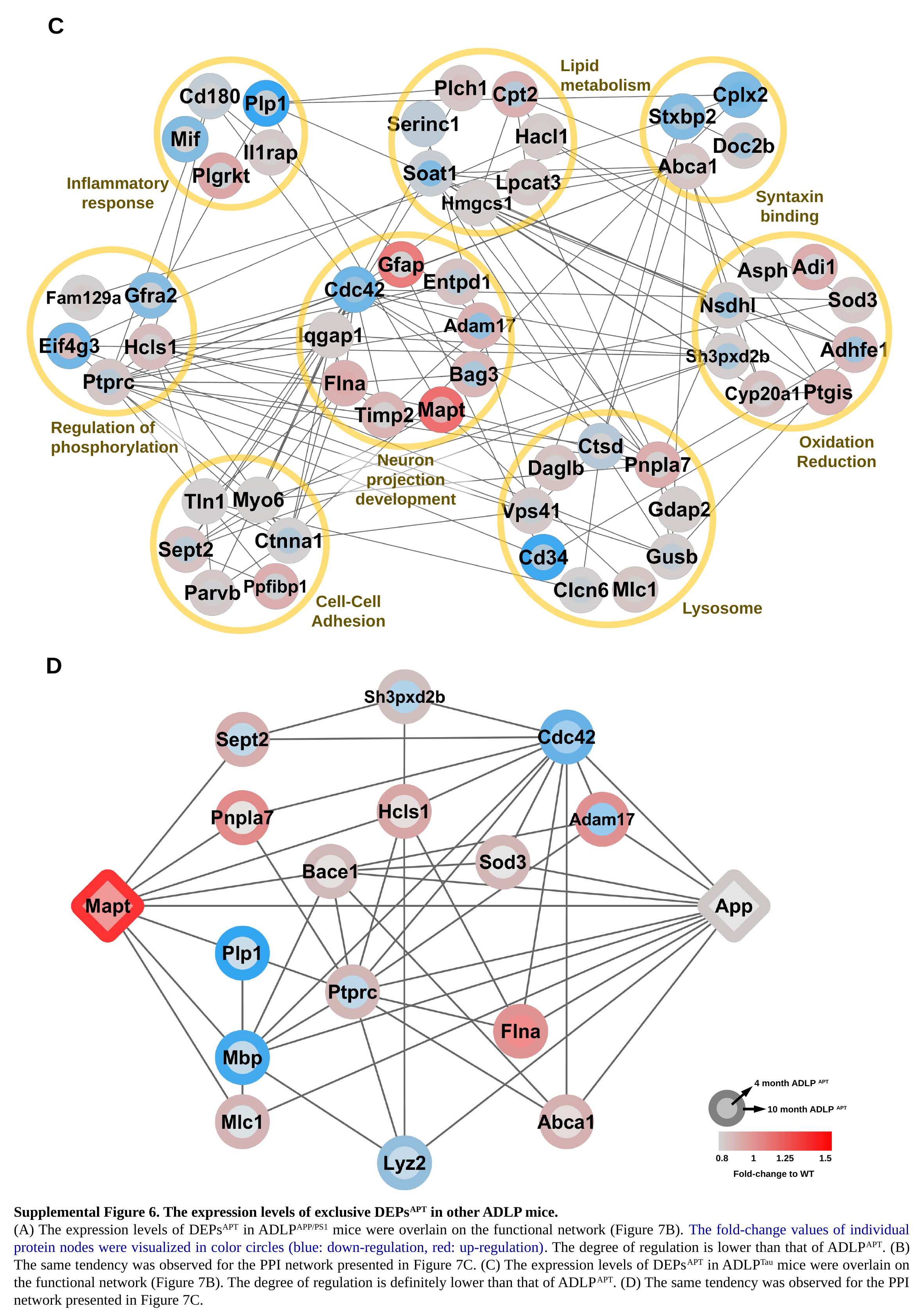

C
Lipid metabolism
Inflammatory response
Syntaxin binding
Regulation of phosphorylation
Oxidation
Reduction
Neuron projection development
Cell-Cell
Adhesion
Lysosome
D
4 month ADLP APT
10 month ADLP APT
0.8 1 1.25 1.5
Fold-change to WT
Supplemental Figure 6. The expression levels of exclusive DEPsAPT in other ADLP mice.
(A) The expression levels of DEPsAPT in ADLPAPP/PS1 mice were overlain on the functional network (Figure 7B). The fold-change values of individual protein nodes were visualized in color circles (blue: down-regulation, red: up-regulation). The degree of regulation is lower than that of ADLPAPT. (B) The same tendency was observed for the PPI network presented in Figure 7C. (C) The expression levels of DEPsAPT in ADLPTau mice were overlain on the functional network (Figure 7B). The degree of regulation is definitely lower than that of ADLPAPT. (D) The same tendency was observed for the PPI network presented in Figure 7C.

## Slide 12
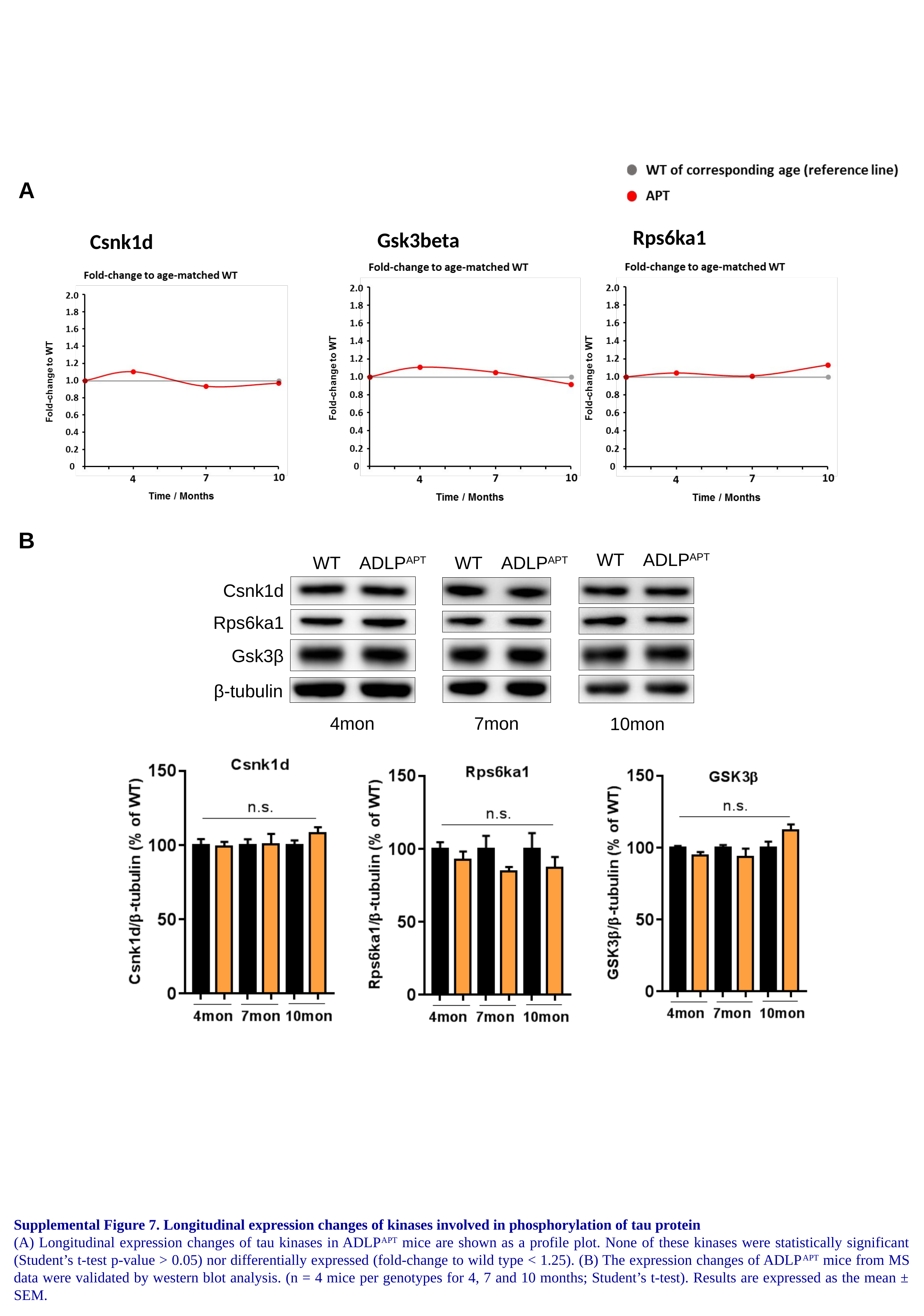

A
Rps6ka1
Gsk3beta
Csnk1d
B
WT ADLPAPT
WT ADLPAPT
WT ADLPAPT
Csnk1d
Rps6ka1
Gsk3β
β-tubulin
4mon
7mon
10mon
Supplemental Figure 7. Longitudinal expression changes of kinases involved in phosphorylation of tau protein
(A) Longitudinal expression changes of tau kinases in ADLPAPT mice are shown as a profile plot. None of these kinases were statistically significant (Student’s t-test p-value > 0.05) nor differentially expressed (fold-change to wild type < 1.25). (B) The expression changes of ADLPAPT mice from MS data were validated by western blot analysis. (n = 4 mice per genotypes for 4, 7 and 10 months; Student’s t-test). Results are expressed as the mean ± SEM.
